# MicroRNA-200c inhibits epithelial-mesenchymal transition, invasion, and migration of lung cancer by targeting HMGB1

**DOI:** 10.1371/journal.pone.0180844

**Published:** 2017-07-20

**Authors:** Po-Len Liu, Wei-Lun Liu, Jia-Ming Chang, Yung-Hsiang Chen, Yu-Peng Liu, Hsuan-Fu Kuo, Chong-Chao Hsieh, Yu-Sian Ding, Wei-Wei Chen, Inn-Wen Chong

**Affiliations:** 1 Department of Respiratory Therapy, College of Medicine, Kaohsiung Medical University, Kaohsiung, Taiwan; 2 Department of Intensive Care Medicine, Chi Mei Medical Center, Liouying, Tainan, Taiwan; 3 School of Medicine, College of Medicine, Fu Jen Catholic University, New Taipei, Taiwan; 4 Preclinical Animal Pharmacology Testing Center, National Research Project for Biopharmaceuticals, New Taipei, Taiwan; 5 Department of Pharmacology, Institute for Drug Evaluation Platform, Development Center for Biotechnology, New Taipei, Taiwan; 6 Graduate Institute of Integrated Medicine, College of Chinese Medicine, China Medical University, Taichung, Taiwan; 7 Department of Psychology, College of Medical and Health Science, Asia University, Taichung, Taiwan; 8 Department of Genome Medicine, Kaohsiung Medical University, Kaohsiung, Taiwan; 9 Department of Internal Medicine, Kaohsiung Municipal Ta-Tung Hospital, Kaohsiung Medical University Hospital, Kaohsiung Medical University, Kaohsiung, Taiwan; 10 Division of Cardiovascular Surgery, Department of Surgery, Kaohsiung Medical University Hospital, Kaohsiung, Taiwan; 11 Department of Internal Medicine, Kaohsiung Medical University Hospital, Kaohsiung, Taiwan; University of South Alabama Mitchell Cancer Institute, UNITED STATES

## Abstract

MicroRNAs (miRs) play critical roles in cancer development, proliferation, epithelial-mesenchymal transition (EMT), invasion, and migration through regulating the expression of oncogenes and tumour suppressor genes. Previous studies have indicated that miR-200c acts as a tumour suppressor in various cancers by downregulating high-mobility group box 1 (HMGB1) and thereby suppressing EMT and metastasis. In addition, miR-200c was reported to be downregulated and correlated with poor outcomes in non-small cell lung cancer (NSCLC). However, its functional role in HMGB1 regulation in NSCLC is still unclear. This study aimed to clarify whether miR-200c acts as a tumour suppressor in NSCLC by downregulating HMGB1, which is associated with EMT, invasion, cytoskeleton rearrangement, and migration in vitro and in vivo. In order to demonstrate HMGB1 downregulation by miR-200c, the NSCLC cell line A549 was transfected with miR-200c mimic or inhibitor. The mimic significantly reduced HMGB1 expression and suppressed EMT, invasion, and migration, while the inhibitor generated the opposite effects. Additionally, using xenograft mouse models, we confirmed that HMGB1 overexpression increased tumour EMT. In summary, our results demonstrated that miR-200c could suppress EMT, invasion, and migration of NSCLC cells by downregulating HMGB1.

## Introduction

Lung cancer is the most common malignancy and a leading cause of cancer-related death worldwide. Non-small cell lung cancers (NSCLC), including squamous cell carcinoma, large cell carcinoma, and adenocarcinoma, are the most common types of lung cancer in Taiwan and these malignancies have a low 5-year survival rate compared with many other types of cancer. Notably, mesenchymal-to-epithelial transition (EMT) processes have been found to regulate tumour progression, metastasis, invasion, and drug resistance in NSCLC [1].

High-mobility group box 1 (HMGB1) is a regulator of chromatin structure that can translocate from the cytoplasm into the nucleus and interact with transcription factors, nucleosomes, and histones to regulate gene expression and multiple other processes including DNA repair, differentiation, inflammation, cell death, and EMT [2, 3]. Furthermore, many studies have demonstrated that HMGB1 can promote malignant phenotypes of cancer cells through increasing proliferation, EMT, and metastasis [4, 5]. Clinically, the overexpression of HMGB1 has been significantly associated with a poor survival rate in various cancers [6–8]. HMGB1 mediates critical processes of EMT in colorectal carcinoma [9], gastric cancer [10], breast cancer [11], and airway epithelium cells [12]. However, it is not known whether HMGB1 can regulate EMT and promote tumorigenesis in the lung.

MicroRNAs (miRNAs or miRs) are small endogenous non-coding RNAs, typically containing 18~22 nucleotides, that regulate the expression of their target genes at the post-transcriptional level. MicroRNA-200c (miR-200c), belongs to the microRNA-200 family, and is highly expressed in normal epithelial cells [13, 14]. Previous studies have demonstrated that endogenous miR-200c suppresses EMT by regulating cell adhesion through targeting the E-cadherin transcriptional repressors ZEB1 and ZEB2 [15, 16]. Furthermore, miR200c has also been reported to regulate proliferation, invasion, metastasis, and chemosensitivity in various cancers [17–19]. Nevertheless, it is not known whether miR-200c acts as a tumour suppressor through downregulating HMGB1 in NSCLC.

This study aimed to investigate whether miR-200c exerts tumour suppressor effects in NSCLC in vivo and in vitro via downregulating HMGB1 and thereby reducing EMT, invasion, and migration. Our results indicated that miR-200c attenuated cancer EMT, invasion, and migration through decreasing HMGB1 expression. This finding supports miR-200c as a potential treatment target in NSCLC.

## Materials and methods

### Cell culture

Lung adenocarcinoma cell line culture was performed as described previously [20]. A549 cells (CCL-185^™^, American Type Culture Collection, Manassas, VA, USA) were cultured in F12K medium (Thermo Fisher Scientific, Waltham, MA, USA) supplemented with 100 pg/ml of streptomycin (Sigma-Aldrich, St. Louis, MO, USA), 100 units/ml of penicillin (Sigma-Aldrich), and 5% (v/v) fetal bovine serum (FBS) (Invitrogen, Carlsbad, CA, USA), at 37°C under a 95% air-5% CO_2_ atmosphere. The culture medium was changed every 4 days, and cells passage between 4 and 13 times were used for experiments.

### Tumour sample collection

Tumours and corresponding normal adjacent lung tissues samples were gathered from patients who underwent surgical resection at the Division of Thoracic Surgery, Department of Surgery, Kaohsiung Medical University Hospital between 2012 and 2014. The study was approved by the Ethical Review Board for Research (reference codes KMUH-IRB-20130344 and KMUH-IRB-20120356) at the Kaohsiung Medical University Hospital, Taiwan and a written informed consent was obtained from each patient.

### Immunohistochemical staining

Immunohistochemical staining was performed as described previously [21]. Normal or tumour tissue sections (5 μm thickness) were deparaffinised and incubated with primary antibodies against HMGB1 (1:1000; GeneTex, Irvine, CA, USA), active β-catenin(1:500; Cell Signaling Technology, Danvers, MA, USA), α-SMA (1:2000; Abcam, Cambridge, UK), and vimentin (1:500, Santa Cruz Biotechnology, Dallas, TX, USA) overnight. Negative control samples were incubated with IgG antibody. After washing in phosphate-buffered saline, the sections were incubated with horseradish peroxidase-conjugated secondary antibody for 1.5 h at room temperature and counter-stained with haematoxylin.

### Immunocytochemistry

Immunocytochemical staining analysis was performed as described previously [22]. Millicell^®^ EZ SLIDES (Millipore, Billerica, MA, USA) were used to culture A549 cells. When cultured cells reached 70% confluency, the cells were washed with cold PBS and then fixed with 4% (w/v) paraformaldehyde in PBS at 4°C for 15 min. After fixation and blocking for 1.5 h, the cells were incubated with primary antibodies against HMGB1 (1:200; GeneTex), α-SMA (1:200; Abcam), and vimentin (1:2100; Abcam) overnight at 4°C, then rinsed with PBS and incubated with rhodamine or fluorescein isothiocyanate-conjugated secondary antibodies for 1 h at room temperature. F-actin was stained with a rhodamine-phalloidin toxin kit (R415, Life Technologies, Carlsbad, CA, USA) [23]. Cell nuclei were counter-stained with 4′, 6-diamidino-2-phenylindole. After washing, cells were mounted in VECTASHIELD^®^ mounting medium (Vector Laboratories, Burlingame, CA, USA) and examined under a fluorescent microscope (Leica, Wetzlar, Germany).

### Migration analysis

The migration assay was performed as described previously [20]. To determine the migration ability of A549 cells, IBIDI^*™*^Culture Inserts (IBIDI, Martinsried, Germany) were placed into 35-mm culture dishes and cells were added into the two reservoirs of the same insert at a density of 1 × 10^5^ cells/ml. After 24 h, the insert was removed with caution, creating a gap of 0.5 mm, and cell migration was monitored by bright-field microscopy at specific time points. The cells that had migrated into the denuded area were photographed and cell-covered areas were measured using the WimScratch software program (Wimasis, Munich, Germany). The experiments were performed in triplicate.

### Cell invasion assay

Cell invasion was assessed by a modified Matrigel^®^ Boyden chamber assay using Bio-Coat^™^ Matrigel^®^ invasion chambers (BD Biosciences, Bedford, MA, USA) according to the manufacturer’s instructions. Cells (1 × 10^5^ per ml) in serum-free medium were seeded onto Matrigel^®^-coated filters, and 5% (v/v) FBS was added to the lower chambers as a chemoattractant. After incubation for 24 h, the membranes were washed briefly with PBS and the upper side of the membrane was wiped gently with a cotton ball. The cells that invaded the lower side of the membrane were removed by Tris-EDTA buffer (10 mM Tris-HCl, pH 8, 0.1 mM ethylenediaminetetraacetic acid) and counted.

### Quantitative real-time PCR

Quantitative real-time PCR was performed as described previously [20]. Total RNA (2 μg) was reverse-transcribed using the SuperScript^®^ First-Strand Synthesis System for real time PCR (Invitrogen), and miRNA was extracted using a miRNA extraction kit (Life Technologies). The resultant cDNA, diluted 1:10, was used as a template to quantify the relative content of miRNA by real-time TaqMan^®^ PCR (LightCycler^®^ FastStart DNA Master SYBR^®^ Green I, Roche, Basel, Switzerland), and cDNA diluted 1:5 was used as a standard. The following primers obtained from Integrated DNA Technologies (Coralville, IA, USA) were used: HMGB1 forward: 5′- GTT CAA GGA TCC CAA TGC AC -3′, reverse: 5′- GAT TTT TGG GCG ATA CTC AGA -3′; CTNNB forward: 5′- CCA GCT CTC TCT TCA GAA CAG -3′, reverse: 5′- GGG TCC ATA CCC AAG GC -3′; ACTA2 forward: 5′- GAC AAT GGC TCT GGG CTC TGT AA -3′, reverse: 5′- ATG CCA TGT TCT ATC GGG TAC TT 3′; GAPDH forward: 5′-AGC CAC ATC GCT CAG ACA-3′, reverse: 5′-GCC CAA TAC GAC CAA ATC C-3′. Primer pairs for the amplification of U6 (P01183321) and miR-200c (P01420724) were obtained from Life Science Technology (MDBio). Relative mRNA expression was calculated by normalising the target mRNA levels to those of house-keeping genes (GAPDH and U6) and the data were compared among treatment groups by the ^ΔΔ^CT method.

### Cell transfection with HMGB1 siRNA and miRNA-200c mimic and inhibitor

Cell transfections with HMGB1 siRNA and miR-200c mimic and inhibitor were performed as described previously [24]. A549 cells were transfected with HMGB1 siRNA (AM16106; Life Technologies), miR-200c-3p mimic (MC11714; Life Technologies) or miR-200c inhibitor (MH11714; Life Technologies)using the Lipofectamine^®^ RNAiMAX kit (Thermo Fisher Scientific). Transfection mixtures containing 0.25 ml of Lipofectamine^®^ 2000 (Life Technologies), 25 ml of Opti-MEM^™^ (Life Technologies), and 10 nM siRNA or15 pmol/ml miR-200c inhibitor or mimic were incubated at room temperature for 10 min and then added to A549 cells seeded in 6-well plates in medium containing 10% (v/v) FBS. Cells were harvested after 48 h and analysed for miRNA expression using a miRNA extraction kit (Life Technologies) and quantitative RT-PCR.

### Western blotting analysis

Western blotting was performed as described previously [24]. Cells were washed with PBS and lysed in protein lysis buffer (Bio-Rad, Hercules, CA, USA) containing protease inhibitors. Cytoplasmic protein extracts were separated by sodium dodecyl sulphate-polyacrylamide gel electrophoresis on 10% (v/v) polyacrylamide gels and transferred to polyvinylidene difluoride membranes for 1 h at room temperature. The membranes were incubated overnight at 4°C with primary antibodies against HMGB1 (1:1,000; GeneTex), active β-catenin (1:500; Cell Signaling Technology), α-SMA (1:2,000; Abcam), vimentin (1:500, Santa Cruz Biotechnology), and α-tubulin (1:2,000, Sigma-Aldrich). After incubation with appropriate horseradish peroxidase-labelled secondary antibodies for 1 h at room temperature, protein bands were detected using ECL-Plus reagent (Millipore) and BioMax^®^ MR Film (Kodak, Rochester, NY, USA), and relative protein expression was quantified by densitometry using the ImageQuant^™^ 5.2 software program (GE Healthcare Life Sciences, Philadelphia, PA, USA).

### Construction of human HMGB1 lentiviral vector and cell transduction

For cloning of human HMGB1 (hHMGB1), total RNA was isolated from A549 cells, and cDNA was produced using MMLV reverse transcriptase according to the manufacturer’s manual (Invitrogen). The construction of ahHMGB1 lentiviral vector and transduction of cells were performed as described previously [24]. The hHMGB1 cDNA (GenBank accession numberNM_002128.4) was amplified by PCR using the following primers: forward 5′-GTC CTA CTA GTG CCA CCA TGG GCA AAG GAG ATC CTA A -3′, reverse 5′- CTT ACG CGG CCG CTT ATT CAT CATCATCAT CTT -3′. The hHMGB1 cDNA was cloned into the pCR2.1-TOPO vector (Invitrogen), and subcloned into the pLEX-MCS lentiviral vector between the BamHI and XhoI restriction sites. Lentivirus particles were prepared by co-transfection of HEK293T cells with the hHMGB1pLEX-MCS vector or vehicle plasmid together with the packaging plasmid SPAX2 and the envelope plasmid MD2G using the calcium phosphate precipitation method. Lentivirus-containing medium was collected 48 and 72 h post-transfection, centrifuged, and filtered through a 0.45-μm syringe filter. For lentivirus transduction, cells were incubated with lentivirus-containing medium mixed 1:1 with culture medium and 5 mg/ml polybrene for 4 h. Then, the medium was replaced by complete culture medium and cells were allowed to grow for 3 days. For stable clone selection, lentivirus-transduced cells were treated with 1 mg/ml puromycin.

### Tumorigenesis of HMGB1-overexpressing A549 lung cancer cells

Twelve male 6–8 week-old SCID mice were purchased from BioLasco Company (Taipei, Taiwan) and quarantined for a week. Animals were housed in a specific pathogen-free room with a 12h light/12h dark cycle and 40–70% humidity at 19–25°C. All animals had access to standard rodent diet and water ad libitum. Animals were subcutaneously inoculated (in the flanks) with 0.1 ml PBS containing 1 × 10^7^ of control or HMGB1-overexpressing A549 cells and monitored for tumour growth twice a week using a digital calliper. The CO_2_ was used for animal sacrifice, which is a promising method for euthanasia in animal welfare and most universities in US use this method to sacrifice animals. The tumor tissue was taken after animal sacrifice and there was no need for anesthesia. Mice will be euthanized under those conditions: (a) lose 15% of animal original body weight; (b) tumors reached mean volumes, which represents greater, larger or more than 15% of body weight; (c) interfere with locomotion and ability to access food and water; (d) ulcerate and result in secondary bacterial infections; (e) after completion of the experimental protocol. All the animal operation and welfare was under the instruction of veterinarian and IACUC regulation. Tumour volume was calculated as T (mm^3^) = length (mm) × width (mm^2^)/2. The protocol for the animal study was reviewed and approved by the Development center for Biotechnology Institutional Animal Care and Use Committee (IACUC number: 2015-R501-031-j).

### Field emission scanning electron microscopy (FE-SEM)

The cells were seeded on cover slip (0.17mm thickness) and fixed in 2.5% glutaraldehyde overnight at 4°C. The cells were washed three times by PBS for 10 minutes each, postfixed in 2% osmium tetraoxide (OsO_4_) for 1.5 h at 4°C. The cells were then washed in three times of PBS for 10 min each, dehydrated in ascending grades of alcohol (50%, 75%, 85%, 95% and 100%). dried by the critical point drier (CPD 030, Bal-TEC, Switzerland) for 1 h. The cells were coated in gold and monitored under field emission scanning electron microscopy (Hitachi-8010, Japan) at accelerating voltage of 10–25 KV.

### Statistical analyses

The data are presented as the mean ± standard error of mean and analysed by the analysis of variance test followed by Dunnett’s test. Statistical analysis was performed using SigmaStat version 3.5 (Systat Software Inc., Chicago, IL, USA), and a p value of less than 0.05 was considered statistically significant.

## Results

### HMGB1, α-smooth muscle actin (SMA), vimentin, and β-catenin are overexpressed in human NSCLC tissue

**Fig 1:**
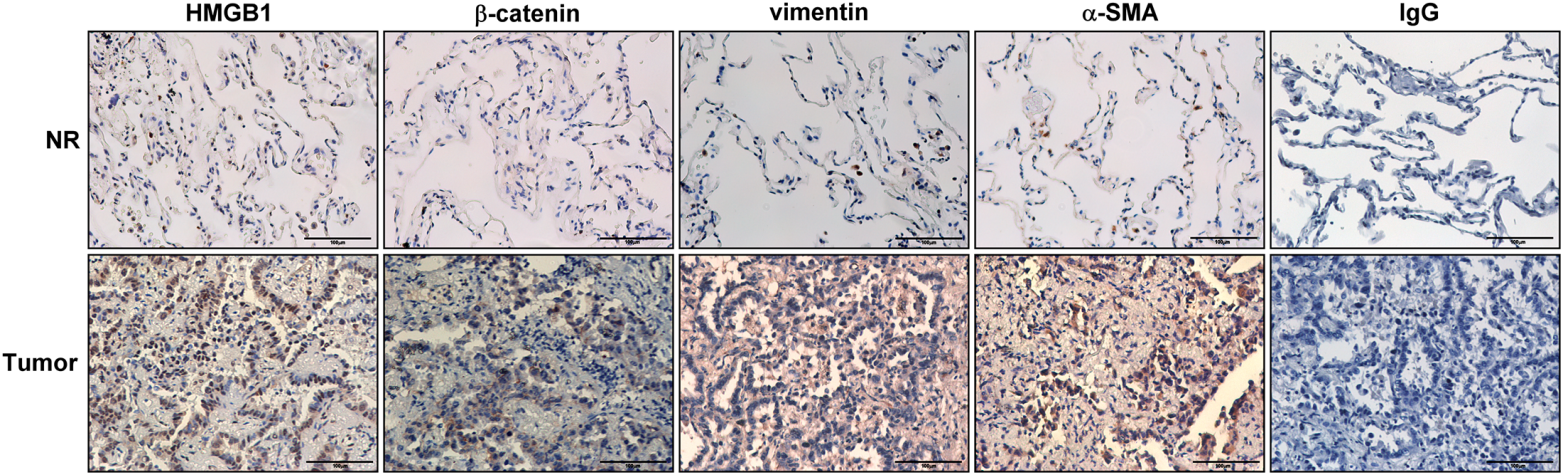
The expression of HMGB1 and epithelial-mesenchymal transition (EMT)-associated proteins in NSCLC patients. Representative photomicrographs are shown that demonstrate increased HMGB1, β-catenin, vimentin, and α-SMA expression in specimens of patients with non—small cell lung cancer (NSCLC). Lung samples (tumor and corresponding normal adjacent lung tissues) were collected and subjected to immunohistochemical staining with antibodies against HMGB1, β-catenin, vimentin, and α-SMA (3,3ʹ-diaminobenzidine staining and hematoxylin counterstaining). For negative controls, the antibody was replaced by control IgG. Scale bar = 100 μm.

Recent studies have verified that HMGB1 is overexpressed in various cancers and regulates EMT, tumour progression, and malignant transformation [8–10]. In order to characterise the expression of HMGB1 and EMT-associated proteins in lung cancer, immunohistochemistry was performed in human NSCLC tissue (Fig 1). The results revealed that the expression levels of HMGB1, α-SMA, vimentin, and β-catenin were significantly increased in tumour tissue when compared with adjacent non-tumour tissue.

### Overexpression or silencing of HMGB1 in lung cancer cells

**Fig 2:**
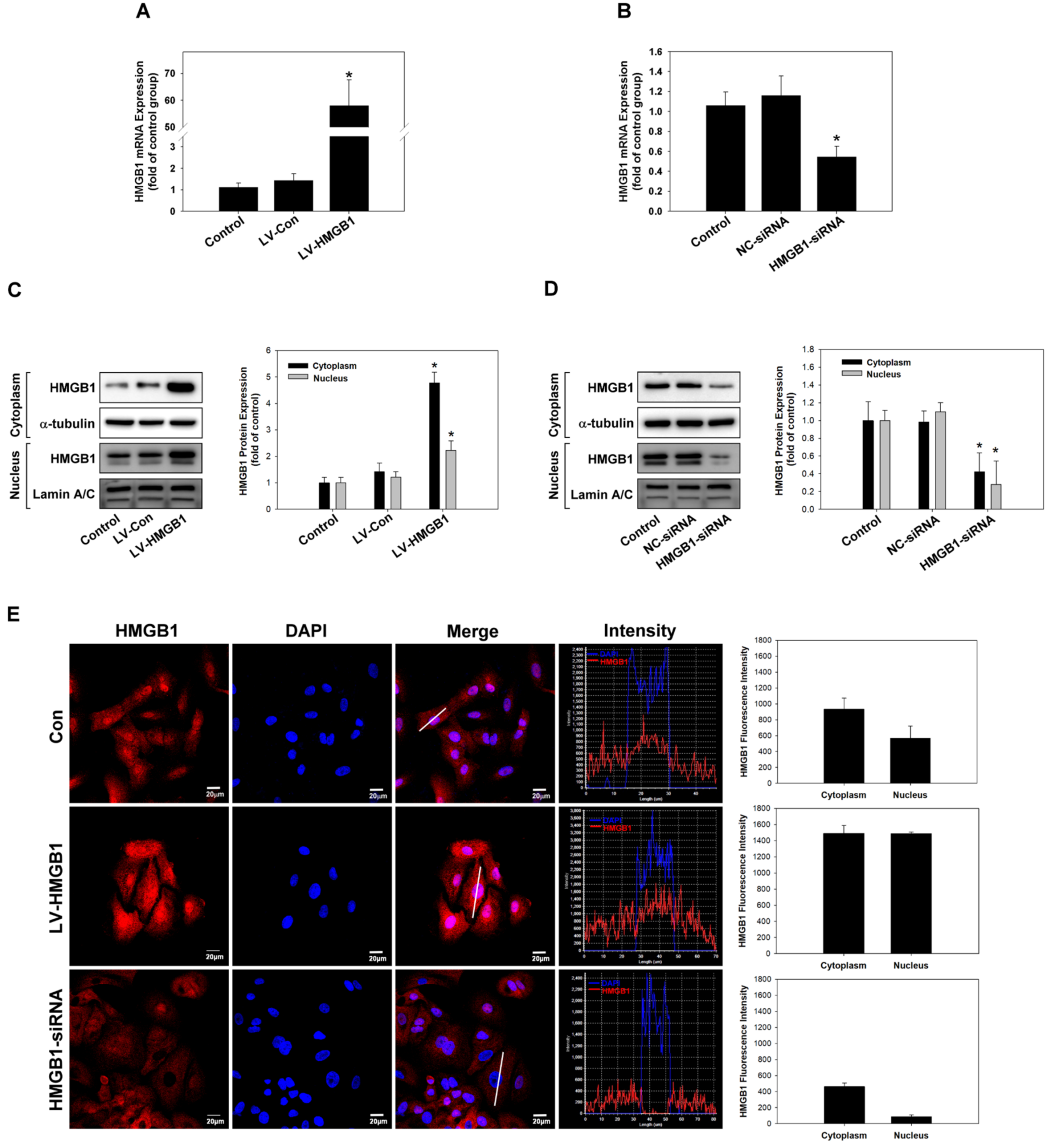
Overexpression or silencing of HMGB1 in lung cancer cells. Overexpression or silencing of HMGB1 expression in A459 NSCLC cells by cloning (LV-HMGB1) or siRNA transfection, respectively, was examined by real-time polymerase chain reaction (PCR) (A and B), western blotting (C and D), and immunocytochemical staining (E). *p <0.05 as compared to the control group. α-Tubulin was used as a loading control. Scale bar = 50 μm.

In order to understand the role of HMGB1 in lung cancer cells, HMGB1 was overexpressed by lentiviral transduction or silenced by siRNA in lung adenocarcinoma A549 cells. The expression levels of HMGB1 mRNA and protein were measured by real-time polymerase chain reaction (PCR) (Fig 2A and 2B), western blotting (Fig 2C and 2D), and immunocytochemistry (Fig 2E). The results showed that we successfully overexpressed or silenced HMGB1 expression in lung cancer cells.

### Overexpression of HMGB1 promotes EMT processes in lung cancer cells

**Fig 3:**
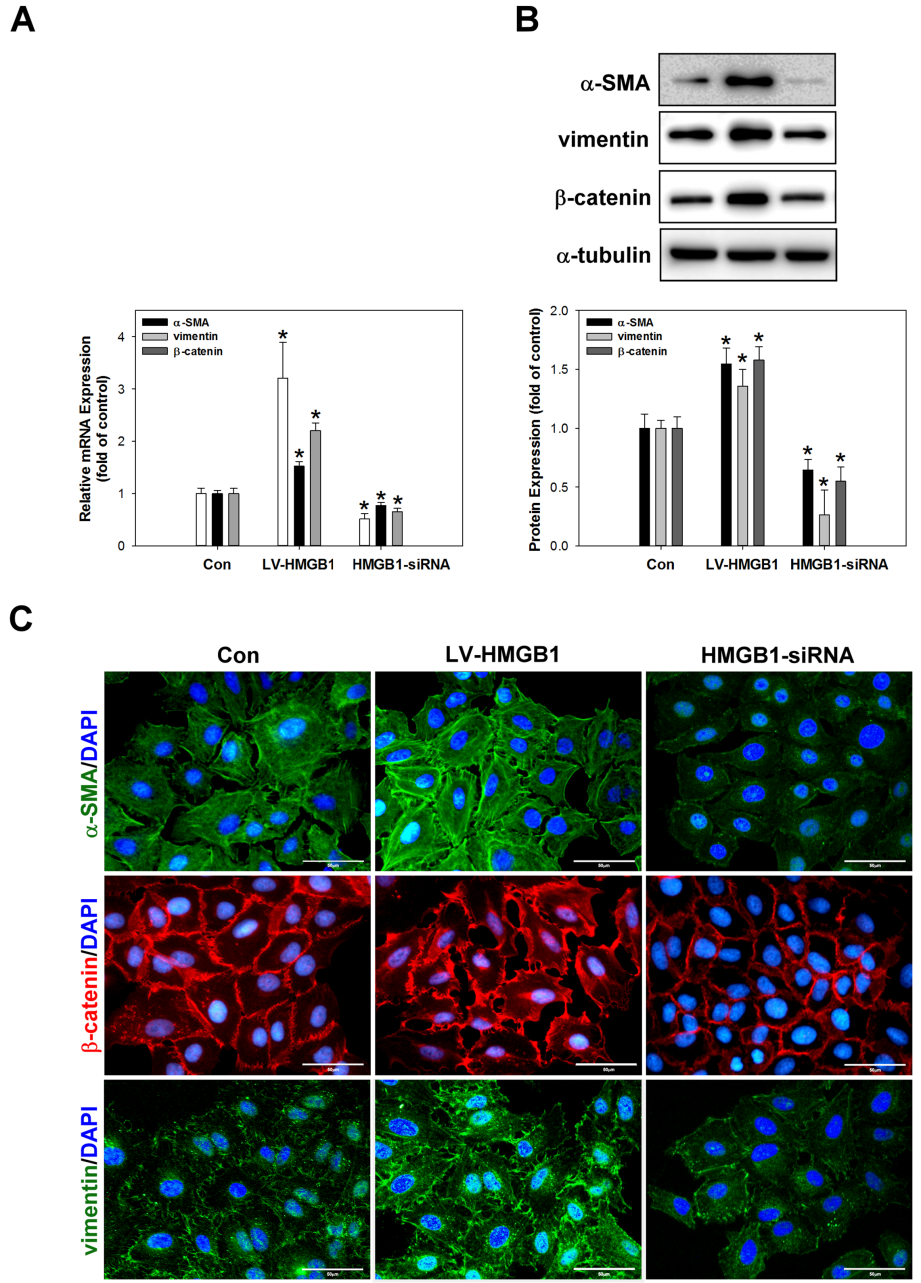
HMGB1 induces the expression of EMT-associated proteins in A549 cells. Overexpression or silencing of HMGB1 in A549 cells regulates α-SMA, vimentin, and β-catenin mRNA and protein expression as determined by real-time PCR (A), western blotting (B), and immunocytochemical staining (C). *p <0.05 as compared to the control group. α-Tubulin was used as a loading control. Scale bar = 50 μm.

Brabletzet al. reported that the loss of E-cadherin and increased nuclear translocation of β-catenin are frequently used as EMT markers [25]. Lee et al. also demonstrated that the metastable or mesenchymal phenotype of lung cancer cells was correlated with the expression of vimentin and α-SMA [26]. In order to investigate the regulation of EMT processes by HMGB1, the expression of the EMT markers α-SMA, vimentin, and β-catenin was analysed by real-time PCR (Fig 3A), western blotting (Fig 3B), and immunocytochemical staining (Fig 3C). The results indicated that the expression of α-SMA, vimentin, and β-catenin was significantly increased by HMGB1 overexpression but attenuated by HMGB1 siRNA transduction was compared to control cells.

### Effects of HMGB1 on migration, invasion, and cytoskeletal rearrangement in lung cancer cells

**Fig 4:**
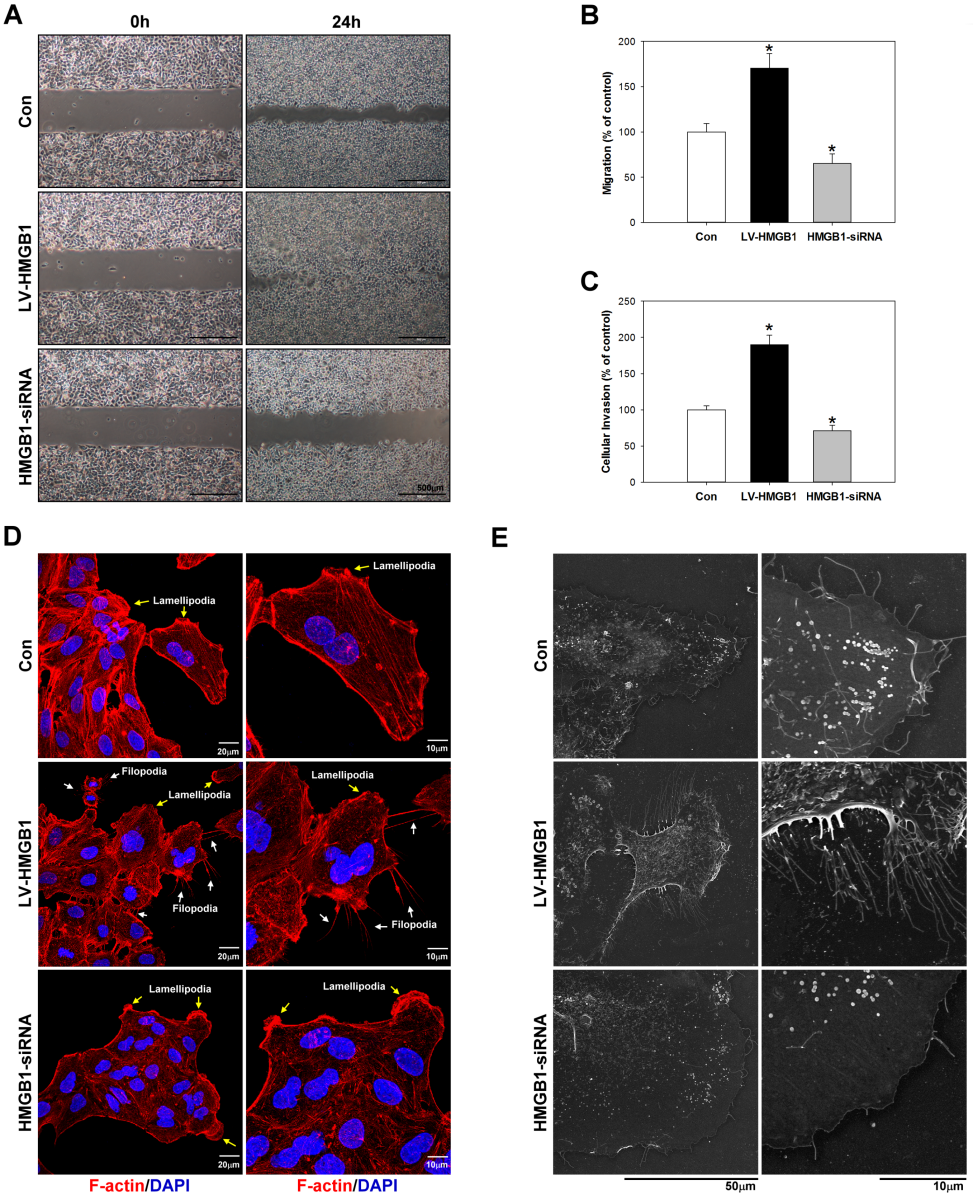
Effects of HMGB1 on migration, invasion, and cytoskeletal rearrangement in lung cancer cells. Overexpression or silencing of HMGB1 was performed in A459 cells for 48h and then the metastatic and invasive abilities of the cells were measured by migration assay (A and B) and invasion assay (C), respectively. Cytoskeletal actin reorganization and cell adhesion dynamics were measured by immunocytochemical staining (D) and field emission scanning electron microscopy (E)*p<0.05 as compared to the control group. α-Tubulin was used as a loading control.

Migration, invasion, and reorganisation of the actin cytoskeleton in lung cancer cells were analysed by Matrigel^®^-coated Boyden chambers migration assay (Fig 4A and 4B), invasion assay (Fig 4C), and filamentous actin (F-actin) immunocytochemical staining (Fig 4D) and field emission scanning electron microscopy (FE-SEM) (Fig 4F), respectively. Reorganisation of the cytoskeleton, including microtubules and actin filaments, plays important roles in cancer cell migration and invasion [27, 28]. As shown in Fig 4D, overexpression of HMGB1 significantly induced F-actin cytoskeletal disorganisation and filopodia rearrangement. Meanwhile, the silencing of HMGB1 expression increased cell-cell adhesion, reduced F-actin expression, and reduced migration and invasion in comparison to control cells. Filopodia are figure-like structures composed of parallel bundles of actin filaments and various actin-associated proteins, and associated with increased migration of metastatic tumor cells [29]. To elucidate HMGB1 enhanced filopodia formation, we examined the formation of filopodial protrusions using FE-SEM (Fig 4E). FE-SEM analysis discovered that small length and thin filopodia protrusions were observed on cell edge in HMGB1-siRNA group (2.5~3.0 μm in length) compared with control (3.0~5.5μm in length) group. In contrast, very long length and fully extended filopodia (10~15μm in length) were observed at the lamellipodial leading edge in LV-HMGB1 group. Our results showed that overexpression of HMGB1 increased the contact area between cells and substrates have an important role in the formation of long and more stable filopodia. The results indicated that HMGB1 could stimulate the migration and invasion of A549 cells by inducing the reorganisation of the actin cytoskeleton.

### miR-200c regulates HMGB1 expression in lung cancer cells

**Fig 5:**
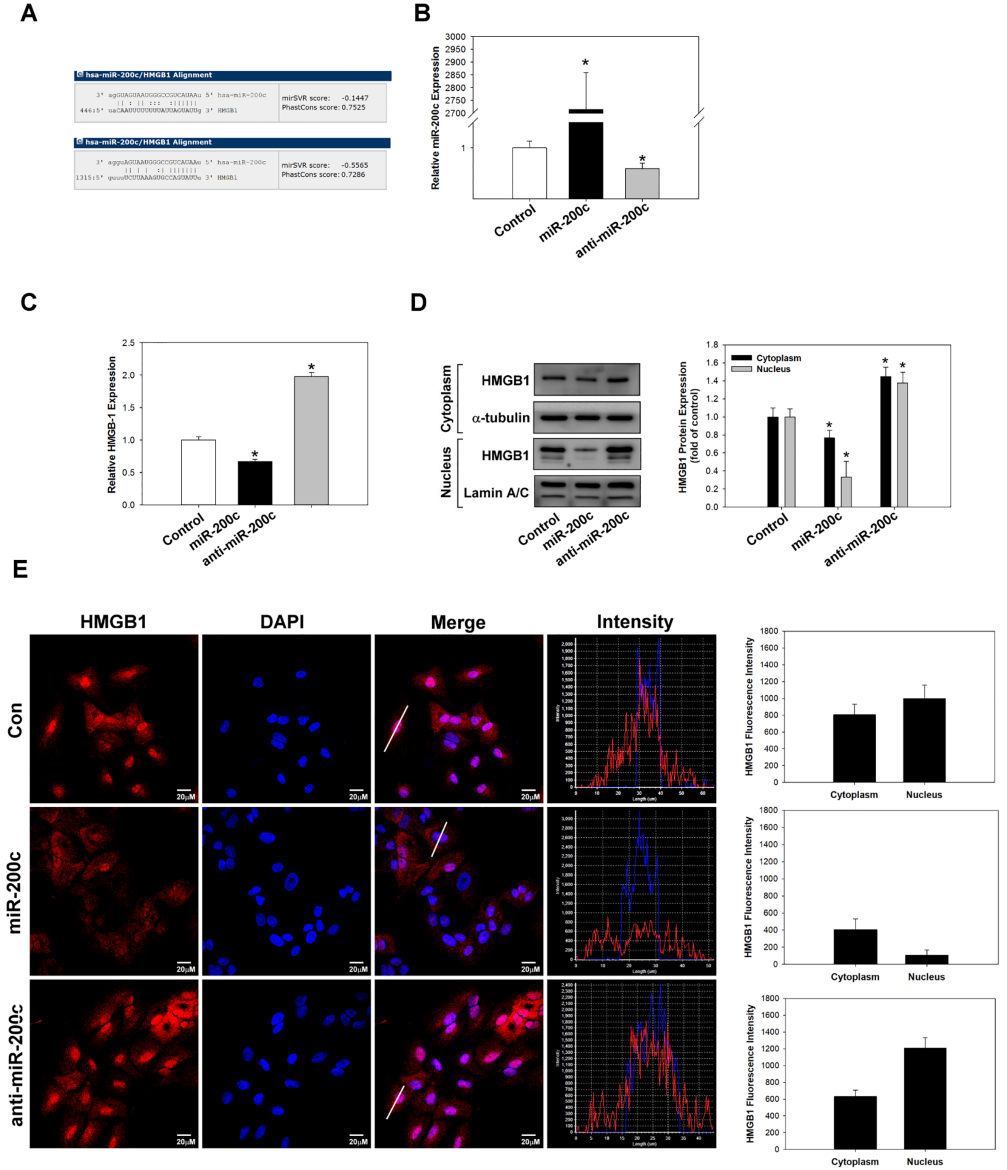
miR-200c regulates HMGB1 expression in lung cancer cells. Alignment of the sequence of miR-200c with that of the 3′untranslated region of HMGB1 showing the putative binding sites (A). Real-time PCR analysis of miR-200c expression in A459 cells after transfection with a miR-200c mimic or inhibitor (B). After transfection, HMGB1 expression was analysed by real-time PCR (C), western blotting (D), and immunocytochemical staining (E). *p <0.05 as compared to the control group. α-Tubulin was used as a loading control.

In various cancers, miR-200c has been demonstrated to be an effective tumour suppressor that inhibits cancer development, proliferation, EMT, therapy resistance, and metastasis [17, 30]. Chang et al. have shown that miR-200c inhibits EMT and metastasis of breast cancer cells by targeting HMGB1 [31], and Jiao et al. have reported that miR-200c inhibits the metastasis of A549 cells by targeting ZEB2, an EMT regulator [32]. However, it remains unknown whether miR-200c can inhibit HMGB1 expression in lung cancer cells. In this study, we transfected miR-200c mimic or inhibitor into A549 cells to identify whether it can inhibit HMGB1 expression and exert anti-tumour effects. To estimate the presence of miRNA-targeted sites in HMGB1, we used the microRNA.org website (http://www.microrna.org/microrna/home.do) and found that HMGB1 (NM_002128) contained potential binding sites for miR-200c (Fig 5A). The expression of miR-200c was measured by real-time PCR analysis (Fig 5B). Moreover, real-time PCR (Fig 5C), western blotting (Fig 5D), and immunocytochemical staining (Fig 5E) were used to evaluate whether HMGB1 expression is regulated by miR-200c in A549 cells. The results indicated that the overexpression of miR-200c significantly reduced HMGB1 expression by binding to its 3′untranslated region. In contrast, the silencing of miR-200c expression increased HMGB1 expression in A549 cells.

### miR-200c inhibits EMT, migration, invasion, and cytoskeletal rearrangement in A549 cells

**Fig 6:**
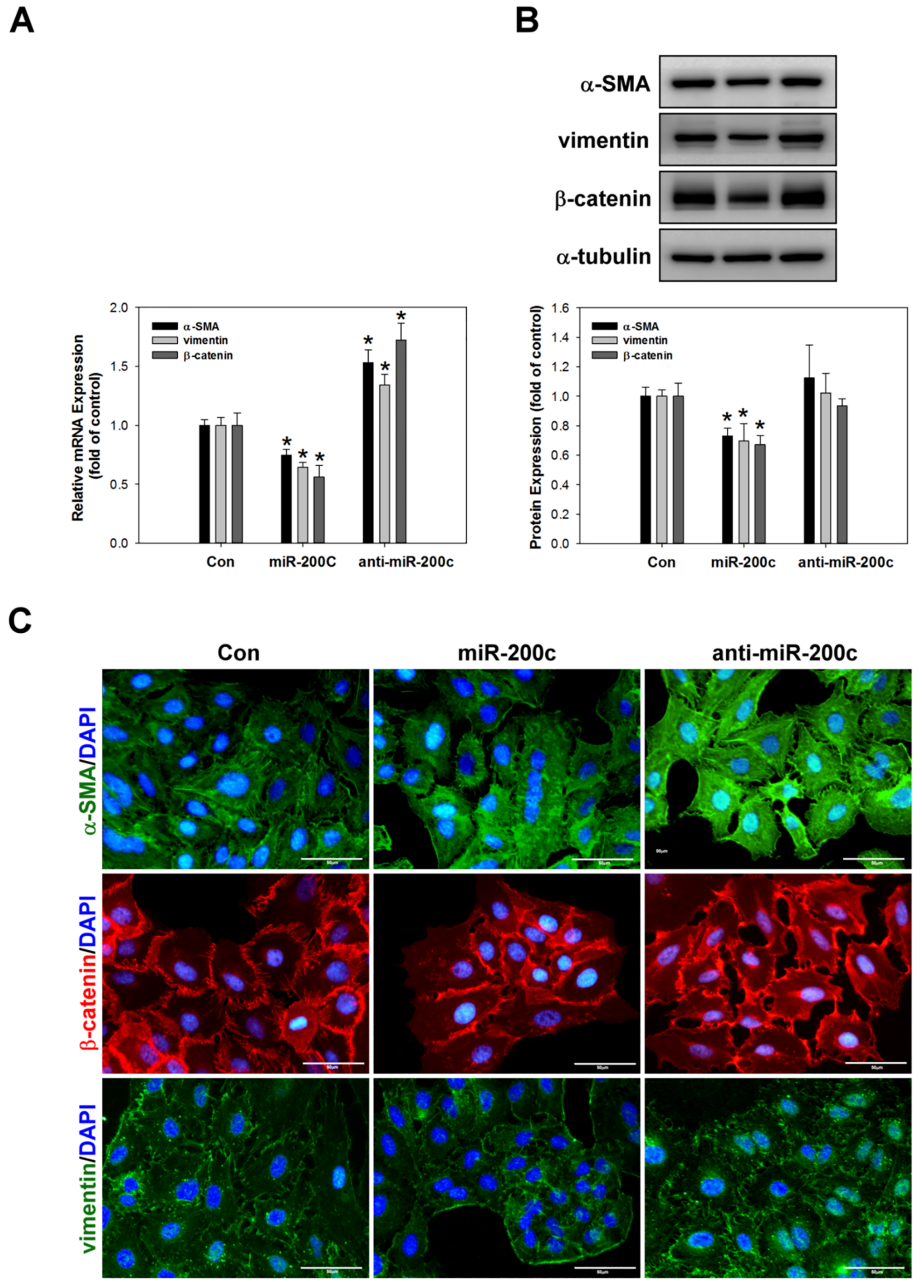
miR-200c suppresses the expression of EMT-associated proteins in A549 cells. A miR-200c mimic or inhibitor was applied to A459 cells for 24h and then the mRNA and protein expression levels of α-SMA, vimentin, and β-catenin were measured by real-time PCR (A), western blotting (B), and immunocytochemical staining (C). *p <0.05 as compared to the control group. α-Tubulin was used as a loading control.

**Fig 7:**
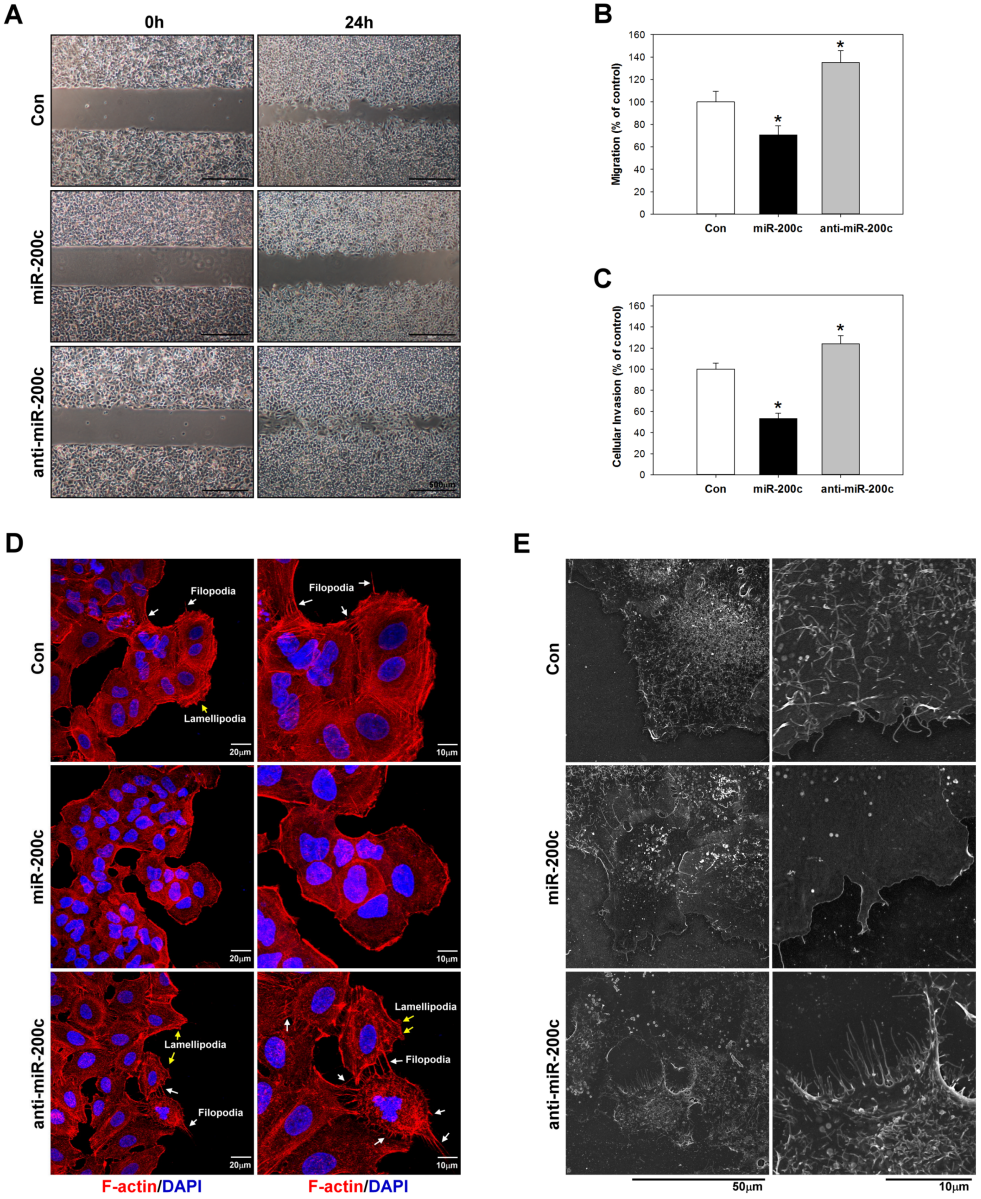
miR-200c inhibits lung cancer cell invasion and metastasis. A miR-200c mimic or inhibitor was applied to A459 cells for 24h and then their metastatic and invasive abilities were measured by migration assay (A and B) and invasion assay (C), respectively. Cytoskeletal actin reorganization and cell adhesion dynamics was measured by immunocytochemical staining (D) and field emission scanning electron microscopy (E). *p <0.05 as compared to the control group.

Previous studies have reported that miR-200c inhibits EMT processes in cancer cells by regulating the expression of EMT markers including E-cadherin, N-cadherin, TCF8/ZEB1, Snail, vimentin, and β-catenin [33, 34]. Therefore, to further evaluate whether miR-200c can inhibit EMT processes in lung cancer, we examined the expression of α-SMA, vimentin, and β-catenin expression by real-time PCR (Fig 6A), western blotting (Fig 6B), and immunocytochemical staining (Fig 6C). The data suggested that miR-200c inhibitsα-SMA and vimentin expression and attenuates the nuclear translocation of β-catenin, indicating that it suppresses EMT processes. Furthermore, Perdigão–Henriques et al. demonstrated that miR-200c suppresses cancer migration and invasion by stabilising actin filaments in lamellipodia and filopodia to promote the epithelial phenotype [35]. In the present study, we evaluated the effect of miR-200c in A549 cells by Matrigel^®^-coated Boyden chambers migration assay (Fig 7A and 7B), invasion assay (Fig 7C), immunocytochemical staining for F-actin (Fig 7D) and FE-SEM (Fig 7E). As shown in Fig 7E, FE-SEM analysis indicated that overexpression of miR-200c was reduced filopodia average number and length (2.0~3.0 μm in length) compared with control (3.0~5.0 μm in length) or anti-miR-200c group (8~10 μm in length). Overexpression of miR-200c significantly decreased filopodia formation and limited the filopodia contact area between cells and substrates.

### miR-200c and HMGB1 expression affect the EMT of NSCLC xenografts in vivo

**Fig 8:**
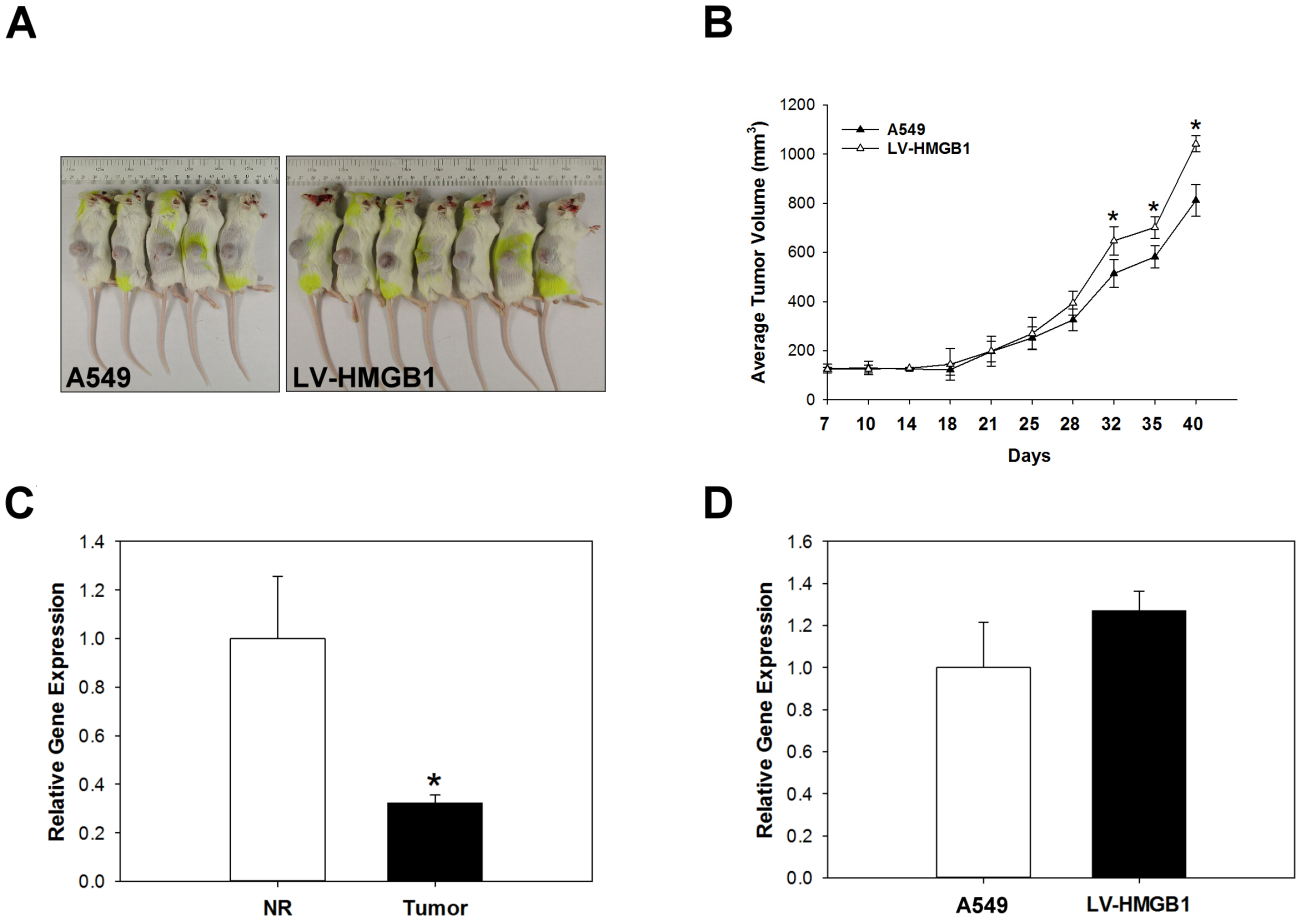
Overexpression of HMGB1 in NSCLC xenografts model. A549 cells and LV-HMGB1 cells were inoculated into the right flank of severe combined immunodeficiency mice. Tumor volume was measured every 3 days with slide calipers starting from day 7, and a growth curve was plotted (A and B). *p <0.05 as compared to the A549 group. Normal/tumour tissue transfected with or without LV-HMGB1were collected and the expression of miR-200c was measured by real-time PCR (C and D). *p <0.05 as compared to the normal (NR) group.

**Fig 9:**
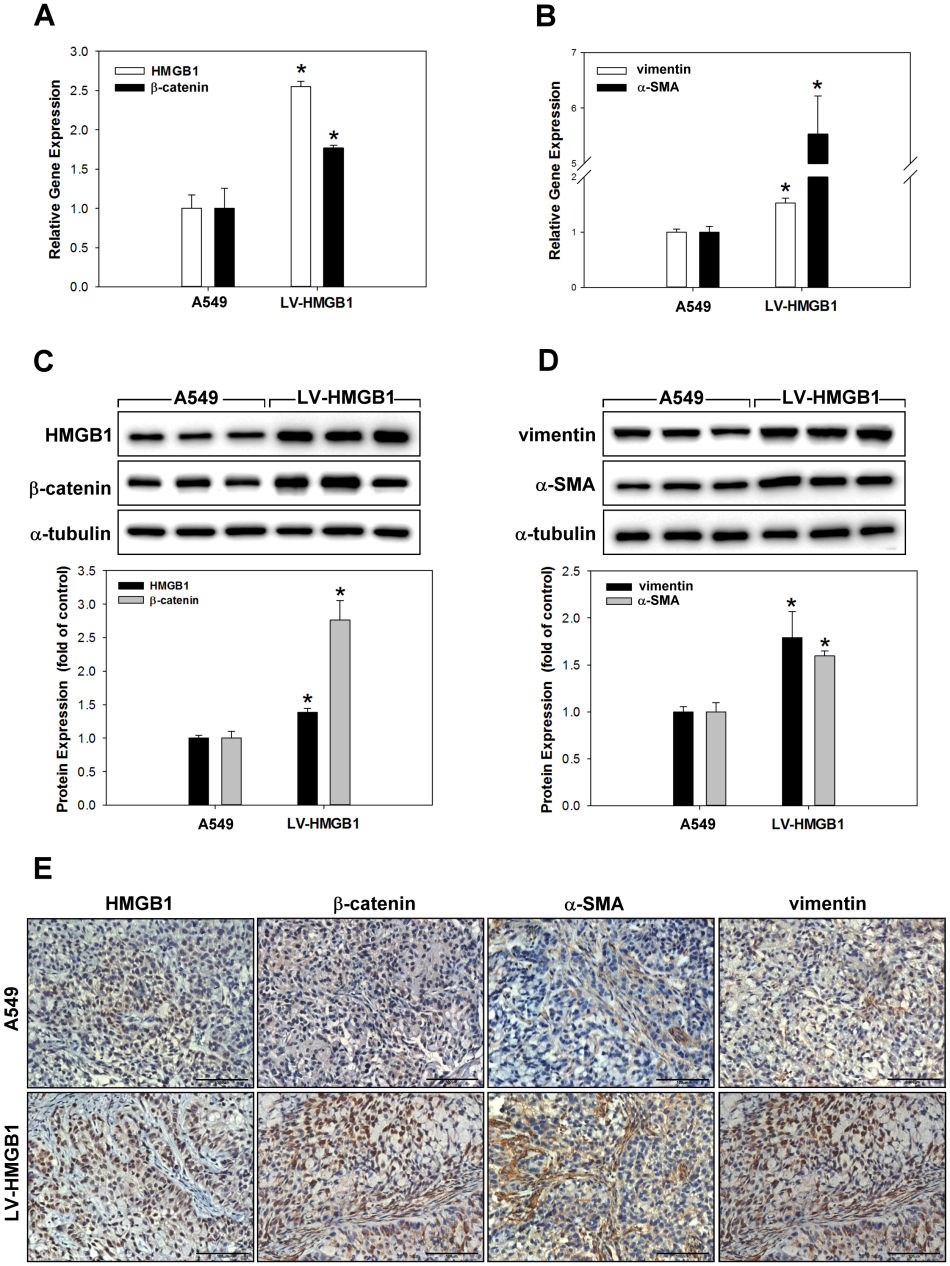
Overexpression of HMGB1 increases the EMT of NSCLC xenografts in vivo. The mRNA and protein expression levels of HMGB1 and EMT markers were measured by real-time PCR (A and B), western blotting (C and D), and immunohistochemistry staining (E). *p <0.05 as compared to the A549 group.

To verify whether HMGB1can increase the tumorigenic potential of lung cancer cells, HMGB1-overexpressing and control A549 cells were subcutaneously transplanted into the flanks of severe combined immunodeficiency (SCID) mice and monitored for tumour growth. Mice were euthanized 40 days post-implantation, the tumor volume A549: 811.7 ± 64.9 mm^3^ and LV-HMGB1: 1042.1 ± 32.5 mm^3^ (Fig 8A and 8B). The expression of miR-200c in normal/tumour tissue transfected with or without LV-HMGB1 was analysed by real-time PCR (Fig 8C and 8D). The data showed that miR-200c was significantly downregulated in tumour tissue compared with normal tissue; in addition, the overexpression of HMGB1 did not directly affect miR-200c expression in NSCLC. To identify whether HMGB1 can regulate EMT processes in vivo, HMGB1 was overexpressed in xenograft lung tissue and the mRNA and protein expression levels of HMGB1, α-SMA, vimentin, and β-catenin were measured by real-time PCR (Fig 9A and 9B), western blotting (Fig 9C and 9D), and immunohistochemical staining (Fig 9E and 9F). The results suggested that the overexpression of HMGB1 increased EMT processes in NSCLC in vivo.

## Discussion

Recent studies have demonstrated that the expression of HMGB1 in NSCLC tumour tissue is markedly increased compared with that in normal tissue. Furthermore, the expression of HMGB1 in lung adenocarcinoma tissue was reported to be significantly higher than that in lung squamous cell carcinoma tissue, and it was associated with patient survival [8, 36]. In this study, we found that a high expression of HMGB1 in lung adenocarcinoma tissues was associated with the expression of cancer EMT markers. This observation indicates that HMGB1 represents an oncogene-like biomarker of lung cancer and is associated with EMT processes.

HMGB1 is a DNA-binding protein that is located in the nucleus and regulates major cellular functions. Recent studies have indicated that the progression of tumorigenesis leads to changes in the intracellular calcium level of cancer cells and results in an increased accumulation of reactive oxygen species. In addition, it has been reported that HMGB1 can be translocated from the nucleus into the cytosol and then released extracellularly via either active or passive secretion processes [37]. Extracellular HMGB1 has been demonstrated to play a critical pro-tumorigenic role through promoting inflammation, EMT, migration, invasion, angiogenesis, and metastasis, while suppressing anticancer immunity [8, 38]. In this study, the overexpression of HMGB1 markedly increased HMGB1 expression in the nucleus, cytosol, and extracellular medium compared with that in non-transfected control cells. Moreover, silencing of HMGB1 expression by siRNA transfection resulted in obvious decreases in the nuclear, cytosolic, and extracellular levels of HMGB1.

The conversion of cells from an epithelial phenotype to a mesenchymal phenotype includes changes in cellular adhesion, structure, morphology, invasion ability, and metastatic capacity [26]. In addition, EMT involvement in oncogenesis or tumour malignancy is controlled by various EMT regulators, including N-myc, ZEB1/2, Snail, Slug, Twist, SRF, and β-catenin [39, 40]. These EMT regulators modulate the expression of various EMT markers in oncogenesis, including E-cadherin, N-cadherin, vimentin, EGFR, α-SMA, EGFR, MMPs, and β-catenin [2, 3]. In this study, the overexpression of HMGB1 was found to induce an increased expression of the EMT markers vimentin, α-SMA, and β-catenin, and it also promoted the nuclear translocation of β-catenin in vitro and in vivo. A recent study reported that after it undergoes nucleus translocation, β-catenin associates with transcription factor 4 (TCF4) and activates the transcription of the EMT-inducing transcription factors ZEB1/2, Snail, Slug, and Twist [41]. The nuclear translocation of β-catenin is widely used as an EMT marker and is associated with a poor prognosis [42]. HMGB1 has been reported to induce EMT through RAGE and the PI3K/AKT/GSK3β/β-catenin signalling pathway [43]. Furthermore, a recent study showed that the treatment of renal cancer cells with recombinant HMGB1 induced vimentin/α-SMA expression and phenotypic change [44]. Taken together with previous reports, our findings suggest that HMGB1 is a key regulator of EMT in lung cancer.

In the present study, HMGB1 overexpression significantly increased the invasion and migration of A549 cells. Overexpression of HMGB1 not only promoted EMT, but also increased cytoskeletal rearrangement in A549 cells. Our data showed that the silencing of HMGB1 expression led to the appearance of a non-polarised cell morphology, and HMGB1 overexpression increased local filopodia expression at the leading edge of cell migration. The cytoplasmic F-actin network was previously reported to be expressed under conditions of stress in renal cancer cells where it became concentrated at the peripheral leading edge of cell migration and regulated cell conformation, mesenchymal phenotype, and cell motility [44]. It has also been demonstrated that filopodia play a crucial role in the cell-cell contact, adhesion, invasion, and migration of epithelial cells. The formation and bundling of actin filaments is essential for filopodia formation both in vitro and in vivo [45]. HMGB1 accelerates the formation of filopodia and there by regulates cancer metastasis [46].

Prior reports have also demonstrated that microRNAs play essential roles in tumorigenesis or tumour suppression in different cancers by targeting the 3′ untranslated region of their target genes. MiR-200c has been shown to have tumour suppressor effects in various cancers [47, 48]. A recent study has demonstrated that miR-200c overexpression significantly accelerates cell cycle arrest at G_0_/G_1_ phase, inhibits cell proliferation, and induces cell apoptosis in A549 cells, possibly through activating the p53/p21 pathway [47]. In A459 cells, the overexpression of miR-200c was found to suppress EMT through downregulating N-cadherin and vimentin while upregulating E-cadherin, and it also significantly reduced cell invasion and migration through inhibiting ZEB2 expression [32]. In this study, the overexpression of miR-200c reduced endogenous HMGB1 expression, suggesting that HMGB1 expression is negatively regulated by miR-200c in A549 cells (Fig 5). Furthermore, the inhibition of miR-200c expression increased EMT, which was similar to the effect of HMGB1 overexpression.

Chang et al. suggested that miR-200c inhibited the invasion and migration of breast cancer cells via targeting the expression of HMGB1 [31]. Our data were consistent with the above study and showed that the overexpression of miR-200c inhibited migration, invasion, and cytoskeletal F-actin rearrangement in A549 cells by downregulating HMGB1. Overall, these data strongly suggest that miR-200c transfection significantly downregulated HMGB1 and suppressed HMGB1-regulated lung cancer EMT and progression. These data support the role of miR-200c as a suppressor of HMGB1 signalling in the lung.

Our study has certain limitations. First, we did not explore the clinical data for the correlation between miR-200c expression and EMT metastasis/invasion; therefore, the results of this study should be applied to human subjects with caution. Second, the study is compromised by use only single cell line. In order to explore the function of HMGB1, stable and sustained overexpression of HMGB1 on lung adenocarcinoma cell lines is required. Actually, we have attempted to perform HMGB1 gene cloning on several NSCLC cell lines including A549, H23 and H441 cells by lentivirus transfection. However, only in the A549 cell line HMGB1 can stable and overexpression. The amplification efficiency of H23 and H441 cell lines were unstable, and the gene amplification was different. Thus, only A549 cells and HMGB1 cloning cells (A549-LV-HMGB1) were used in our study. Third, previous study has showed that HMGB1 promotes the expression of MMP2/9 and A549 invasion activity [49]. On the other hand, the role of MMP8 in lung cancer invasion ability is not clear, González-Arriaga *et al*. have shown that the polymorphism in MMP8 is associated with a decreased lung cancer risk, which can be used as a prognostic marker in lung cancer [50]. The invasion activity of MMP8/9 needs to be further investigated. Fourth, miR200c is also known to target ZEB2, which can inhibit EMT in lung cancer especially A549 cells. How the effects of targeting both HMGB1 and ZEB2 in contributing to the EMT inhibition induced by miR200c need to be further performed. Finally, the metastatic progression could be demonstrated most suitable with tail vein inject model. However, the success rate of the A549 lymph node metastasis in the tail vein inject model is low and the experimental progression of the metastasis is relative long. Thus, animals were subcutaneously inoculated with A549 cells in the present study. Further studies should be performed to confirm the roles of HMGB1 with the tail vein inject model.

## Conclusions

Our results characterised HMGB1 as a potential target of miR-200c and showed that miR-200c may regulate A549 cell EMT, and cancer progression by targeting HMGB1. Therefore, miR-200c may represent a molecular biomarker of NSCLC outcome, and on the other hand, it could be a therapeutic target for the reduction of lung cancer progression. Further studies of HMGB1-regulated signalling pathways implicated in lung cancer EMT and cancer progression are required to validate the potential of miR-200c for use in therapy for lung cancer.

## Supporting information

S1 Fig Overexpression or silencing of HMGB1 in lung cancer cells. S1 is Fig 2C and 2D raw data.
(DOCX)

S2 Fig HMGB1 induces the expression of EMT-associated proteins in A549 cells. S2 is Fig 3B raw data.
(DOCX)

S3 Fig miR-200c regulates HMGB1 expression in lung cancer cells. S3 is Fig 5D raw data.
(DOCX)

S4 Fig miR-200c suppresses the expression of EMT-associated proteins in A549 cells. S4 is Fig 6B raw data.
(DOCX)

S5 Fig Overexpression of HMGB1 increases the EMT of NSCLC xenografts in vivo. S5 is Fig 9C and 9D raw data.
(DOCX)
