# Supplementary material for: MicroRNA-200c inhibits epithelial-mesenchymal transition, invasion, and migration of lung cancer by targeting HMGB1
Source: PLoS One. 2017 Jul 20;12(7):e0180844. doi: 10.1371/journal.pone.0180844 (PMC5519074; doi:10.1371/journal.pone.0180844)

**S1 Fig. Overexpression or silencing of HMGB1 in lung cancer cells.**

**Figure-2C Figure-2D**

Con LV-Con LV-HMGB1 Con NC-siRNA HMGB1-siRNA


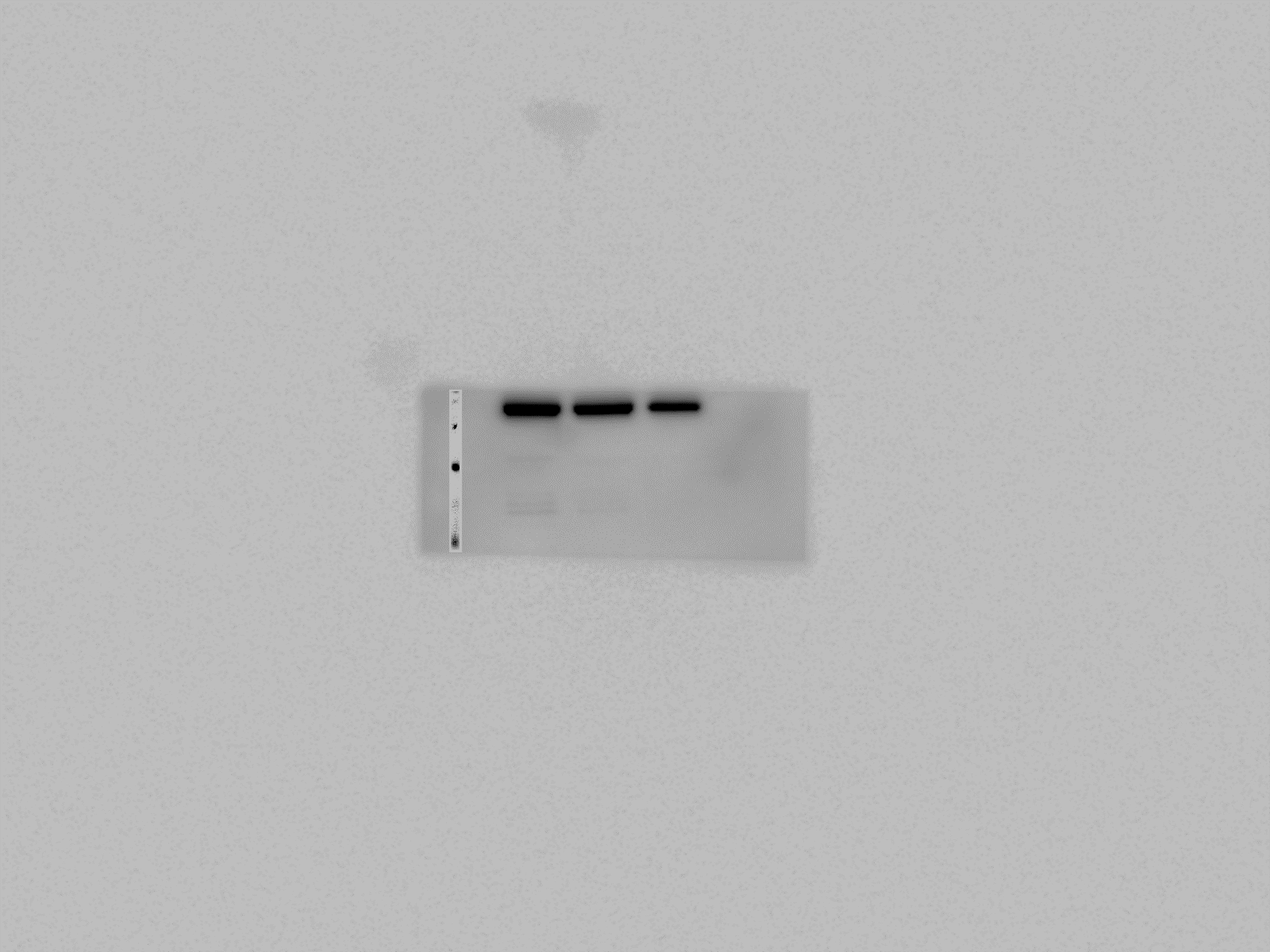

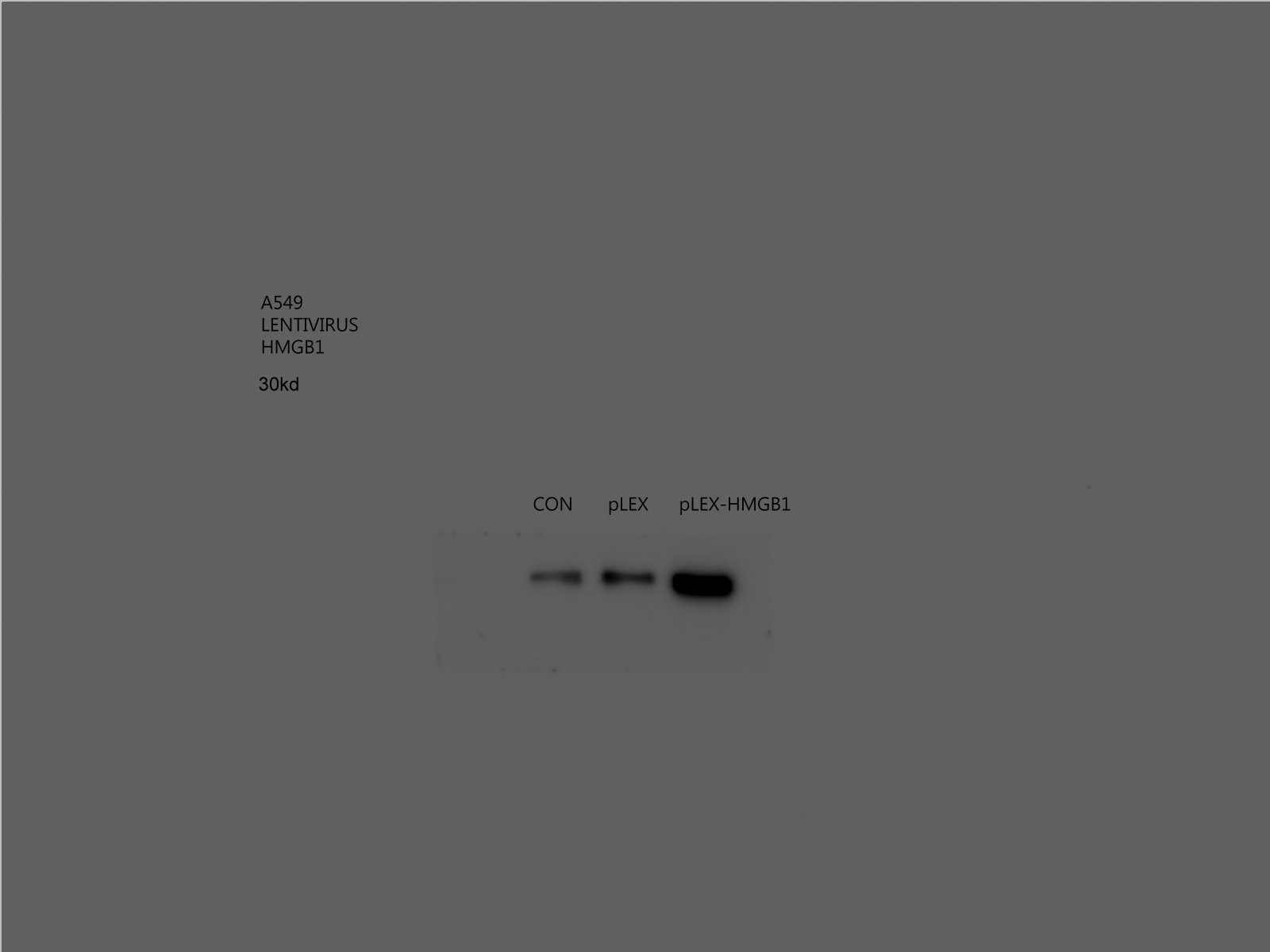


HMGB1

HMGB1


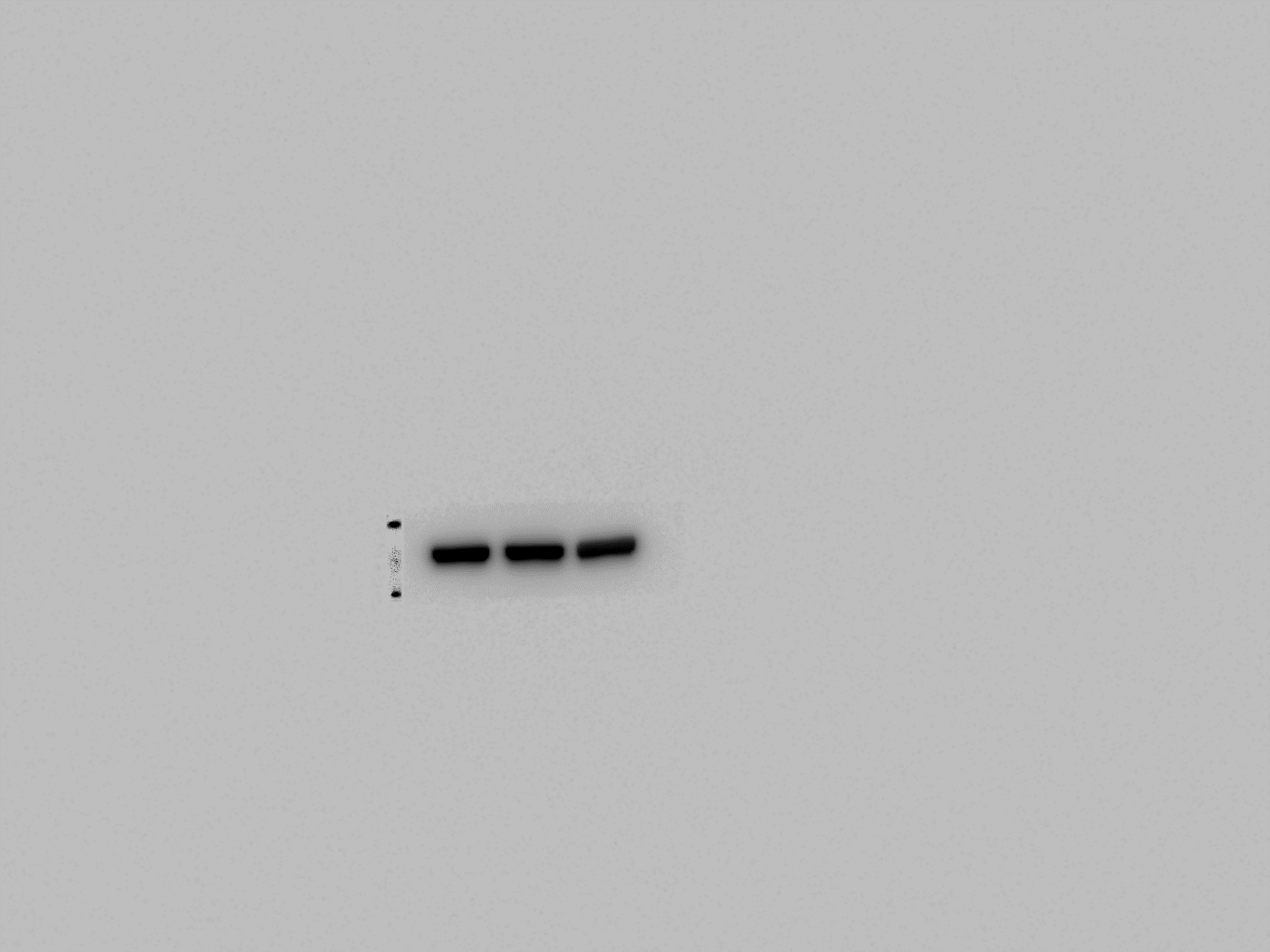

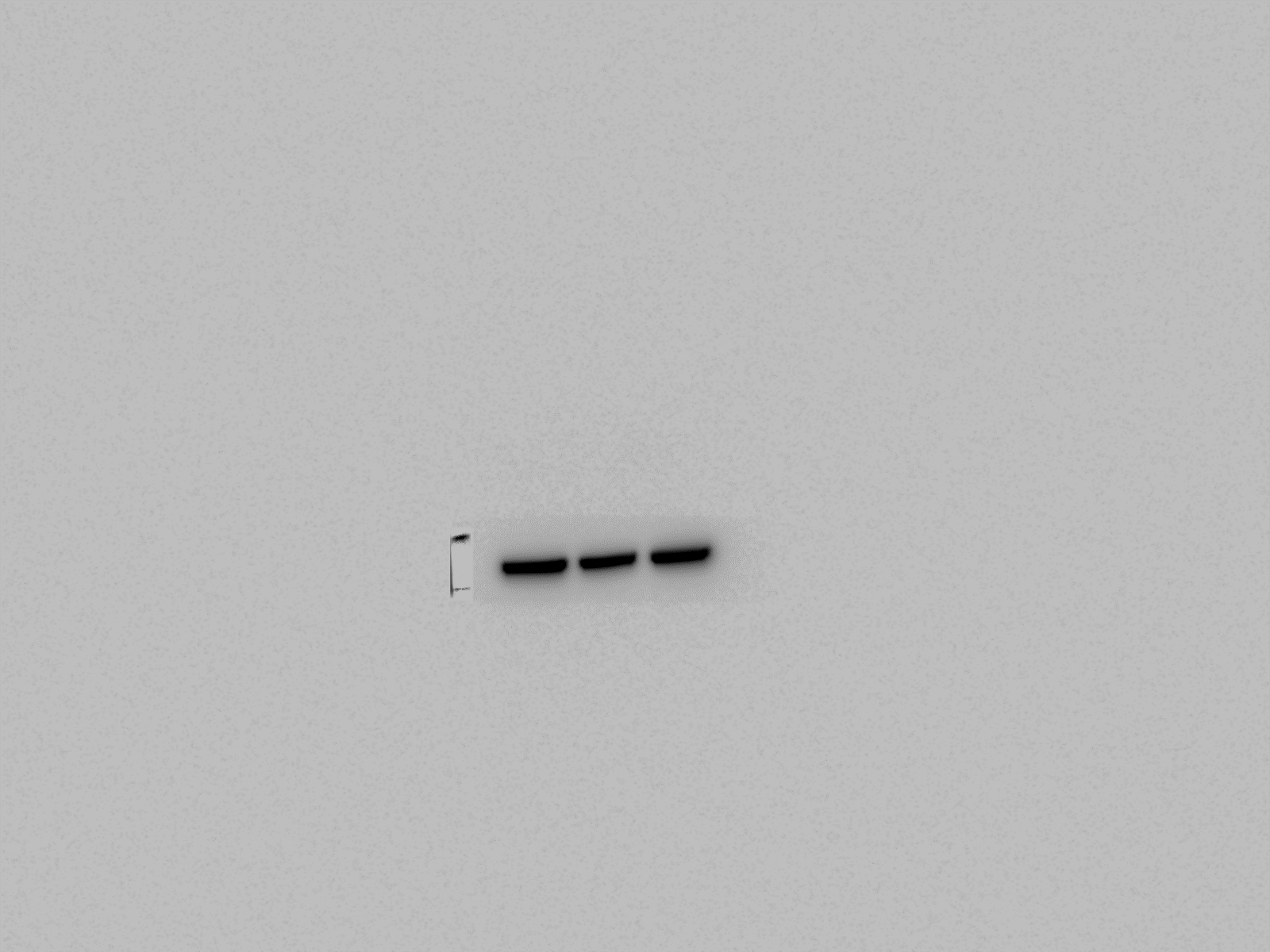


α-tubulin

α-tubulin


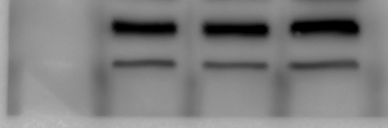

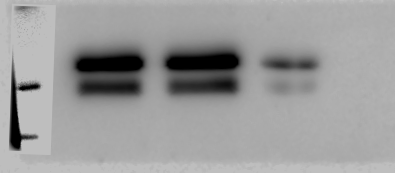

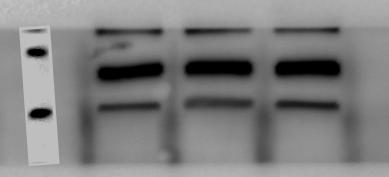


HMGB1

Lamin A/C

Lamin A/C

HMGB1


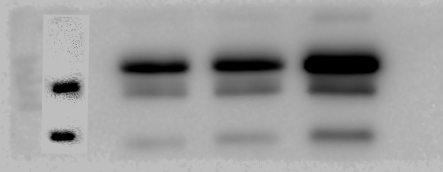

Supplement: S1 Fig — S1 is Fig 2C and 2D raw data. (DOCX) [file pone.0180844.s001.docx]
