# Supplementary figures and images for: MicroRNA-200c inhibits epithelial-mesenchymal transition, invasion, and migration of lung cancer by targeting HMGB1
Source: PLoS One. 2017 Jul 20;12(7):e0180844. doi: 10.1371/journal.pone.0180844 (PMC5519074; doi:10.1371/journal.pone.0180844)

**S2 Fig. HMGB1 induces the expression of EMT-associated proteins in A549 cells.**

**Figure-3B**


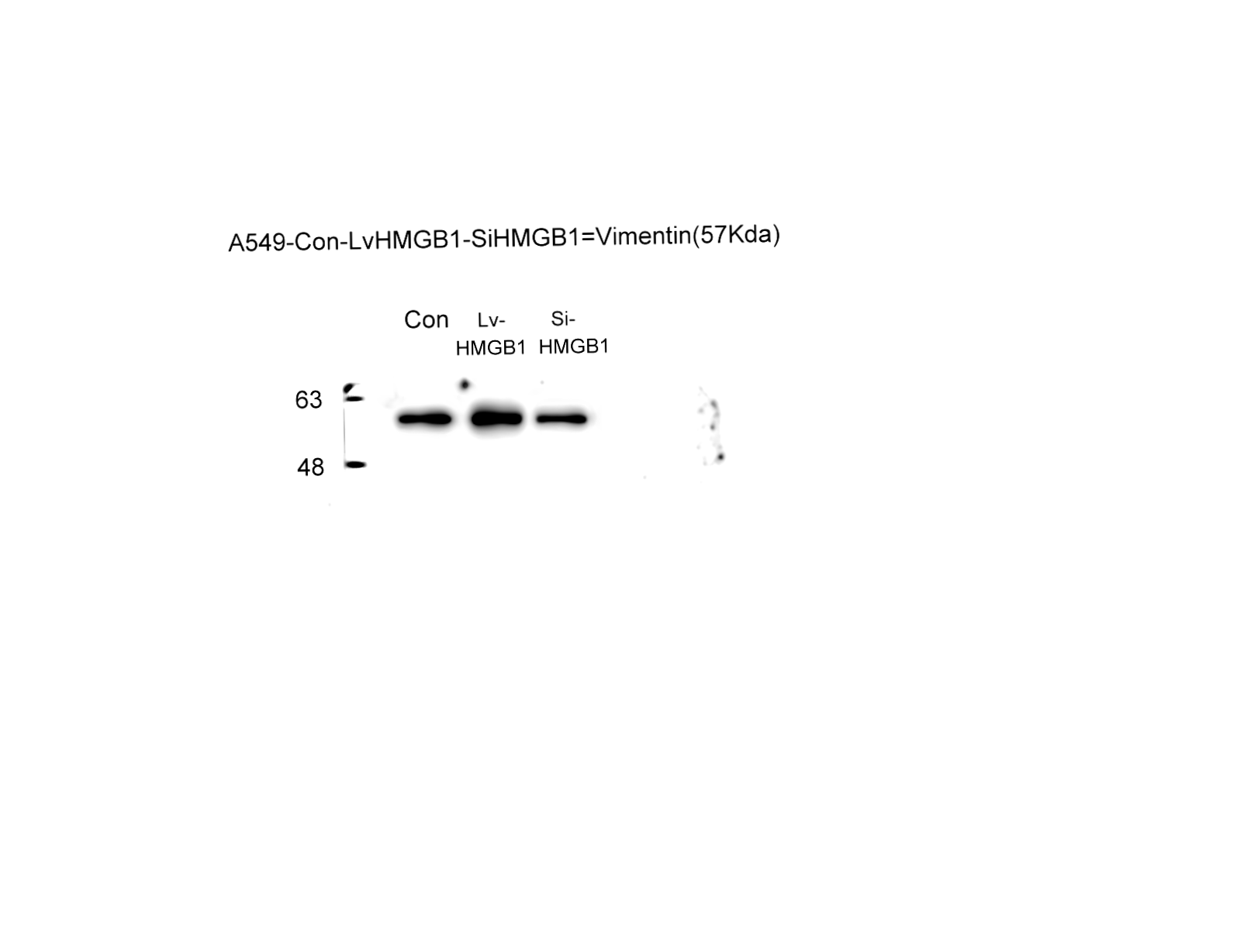


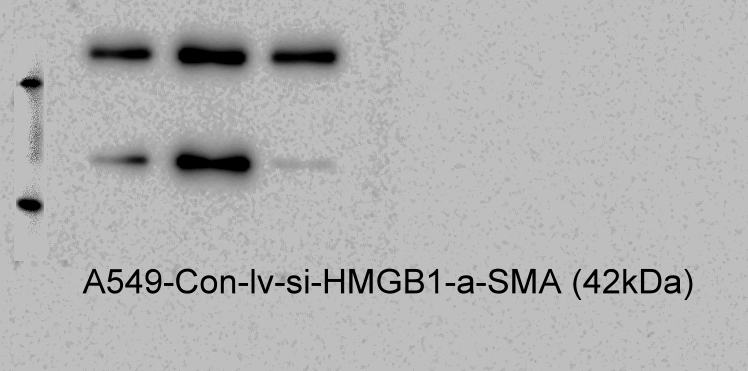


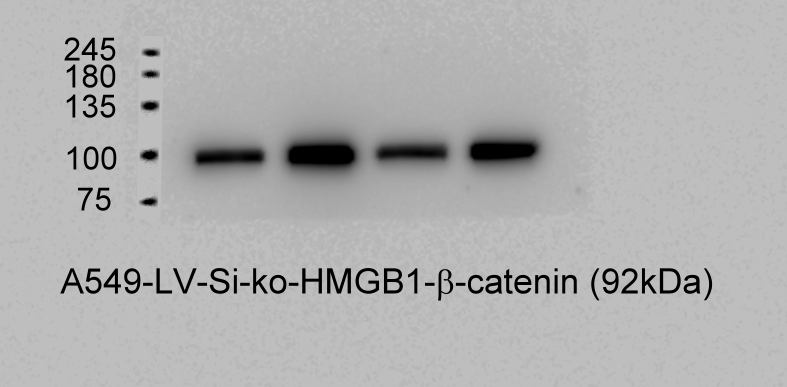


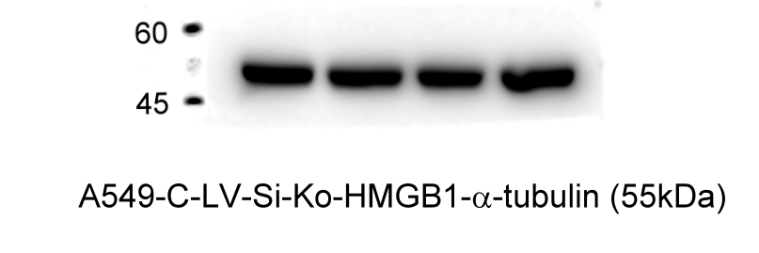


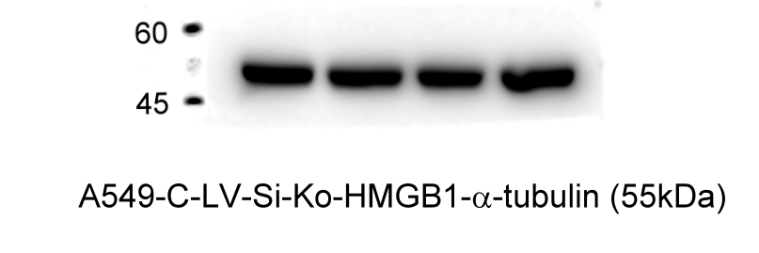

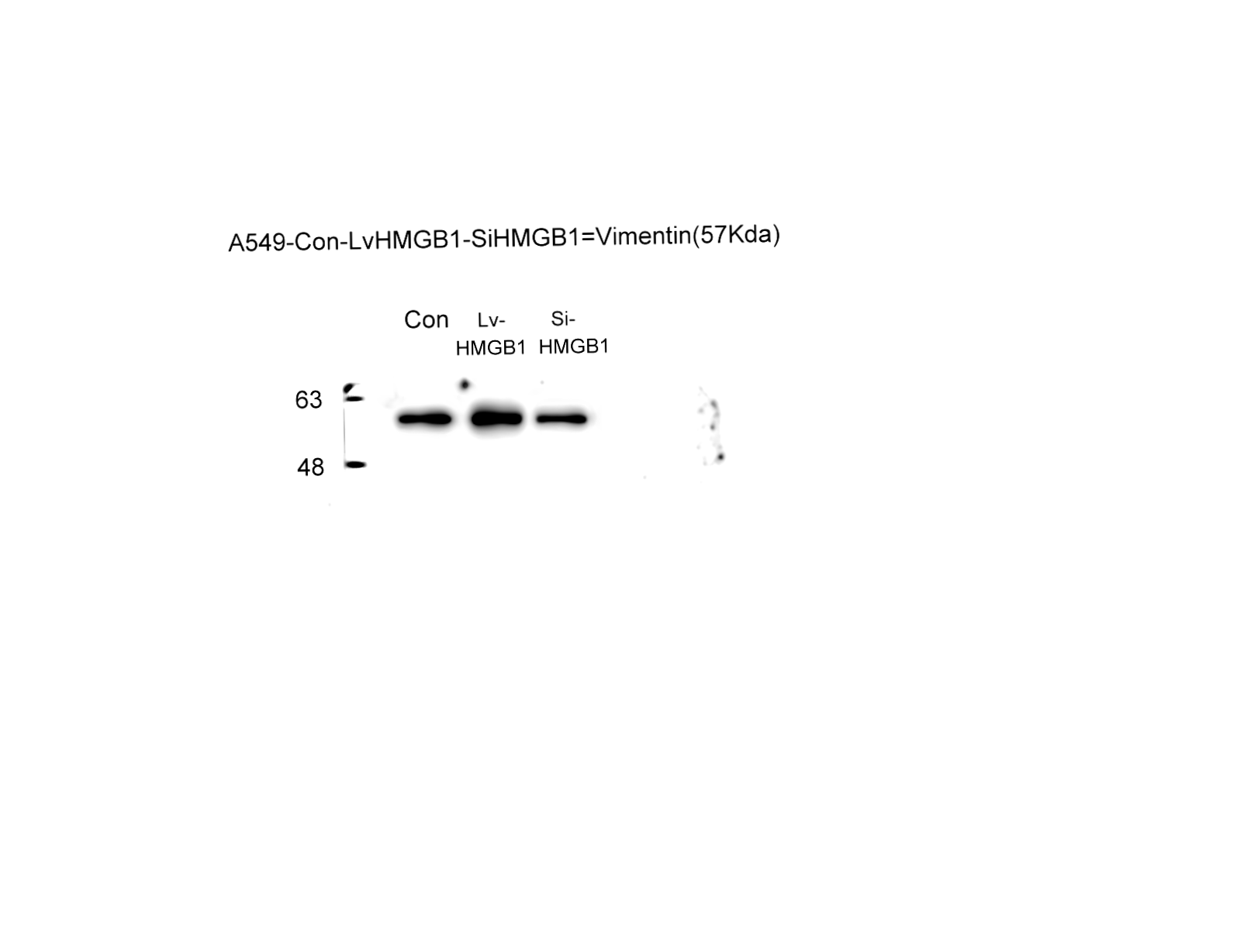

Supplement: S2 Fig — S2 is Fig 3B raw data. (DOCX) [file pone.0180844.s002.docx]
