# Supplementary material for: MicroRNA-200c inhibits epithelial-mesenchymal transition, invasion, and migration of lung cancer by targeting HMGB1
Source: PLoS One. 2017 Jul 20;12(7):e0180844. doi: 10.1371/journal.pone.0180844 (PMC5519074; doi:10.1371/journal.pone.0180844)
Supplement: S3 Fig — S3 is Fig 5D raw data. (DOCX) [file pone.0180844.s003.docx]

**S3 Fig. miR-200c regulates HMGB1 expression in lung cancer cells.**

**Figure-5D**

Con mimic inhibitor


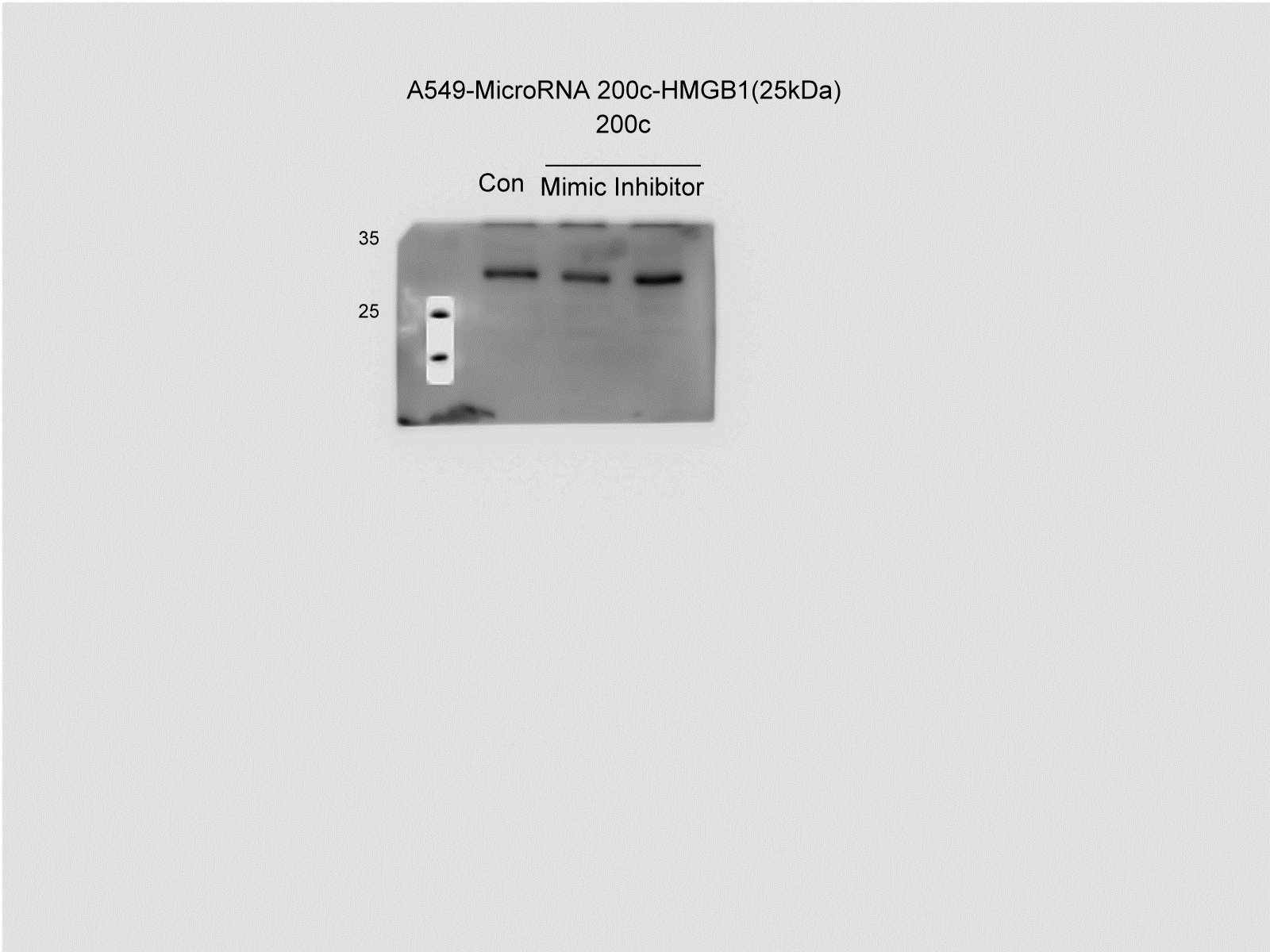


Lamin A/C

HMGB1


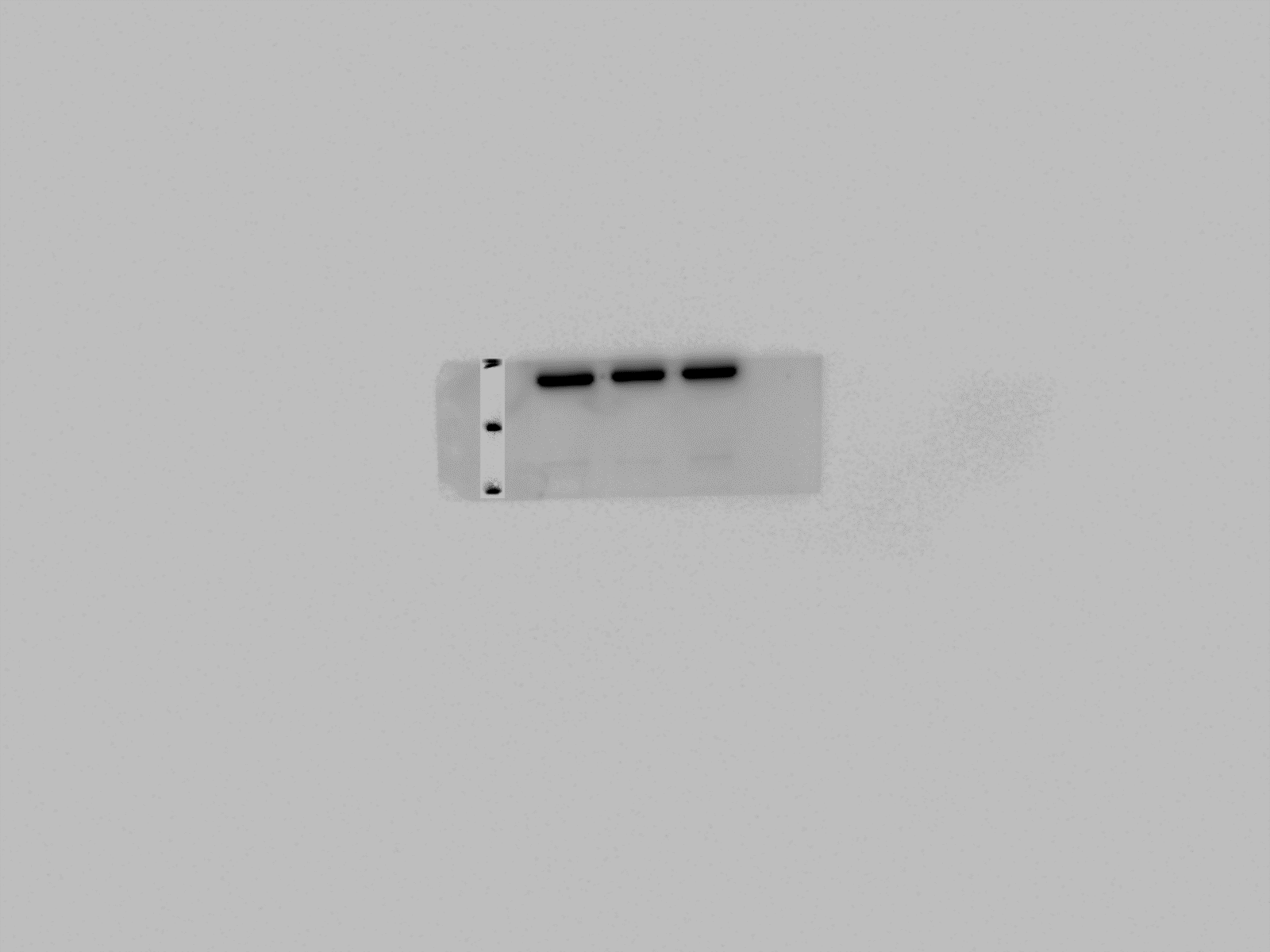

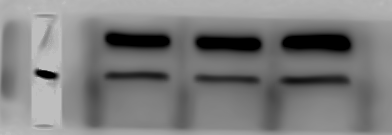

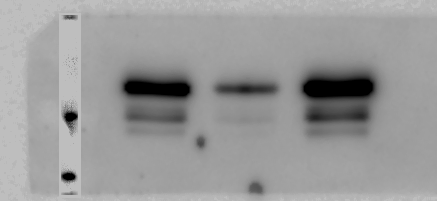


α-tubulin
