# Supplementary material for: MicroRNA-200c inhibits epithelial-mesenchymal transition, invasion, and migration of lung cancer by targeting HMGB1
Source: PLoS One. 2017 Jul 20;12(7):e0180844. doi: 10.1371/journal.pone.0180844 (PMC5519074; doi:10.1371/journal.pone.0180844)
Supplement: S4 Fig — S4 is Fig 6B raw data. (DOCX) [file pone.0180844.s004.docx]

**S4 Fig. miR-200c suppresses the expression of EMT-associated proteins in A549 cells.**

**Figure-6B**

Con miR-200c anti-miR-200c


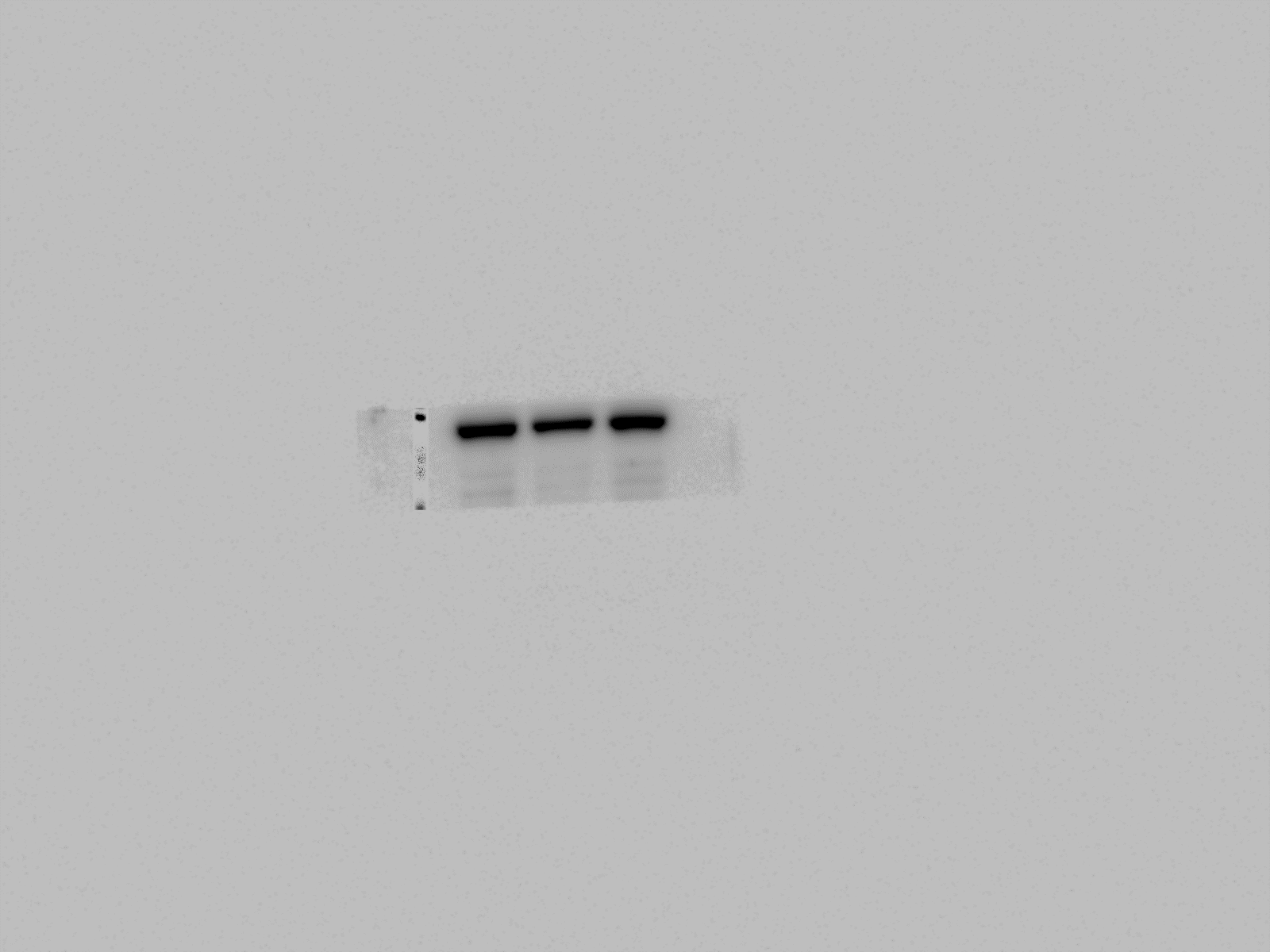


α-SMA


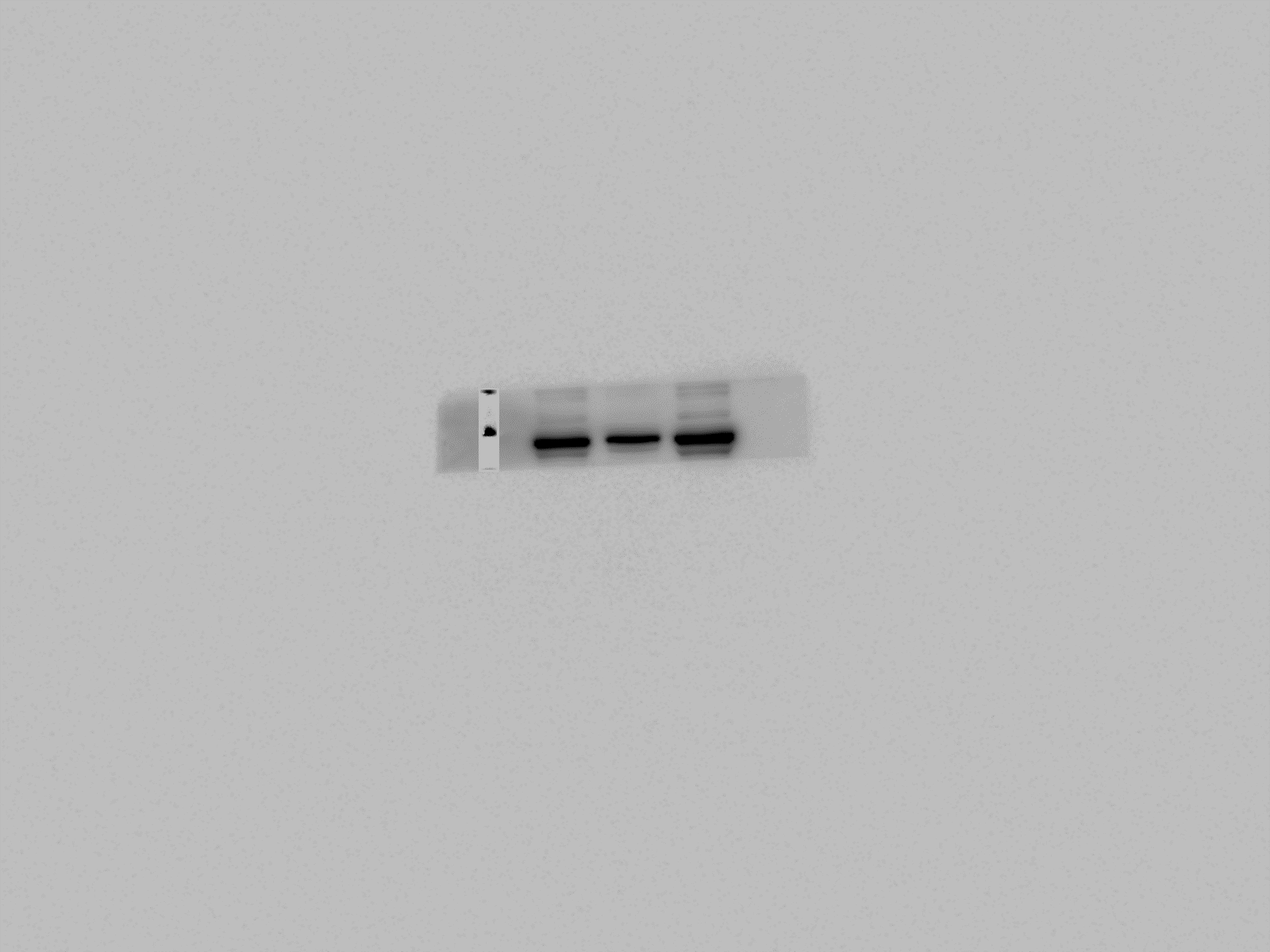


Vimentin


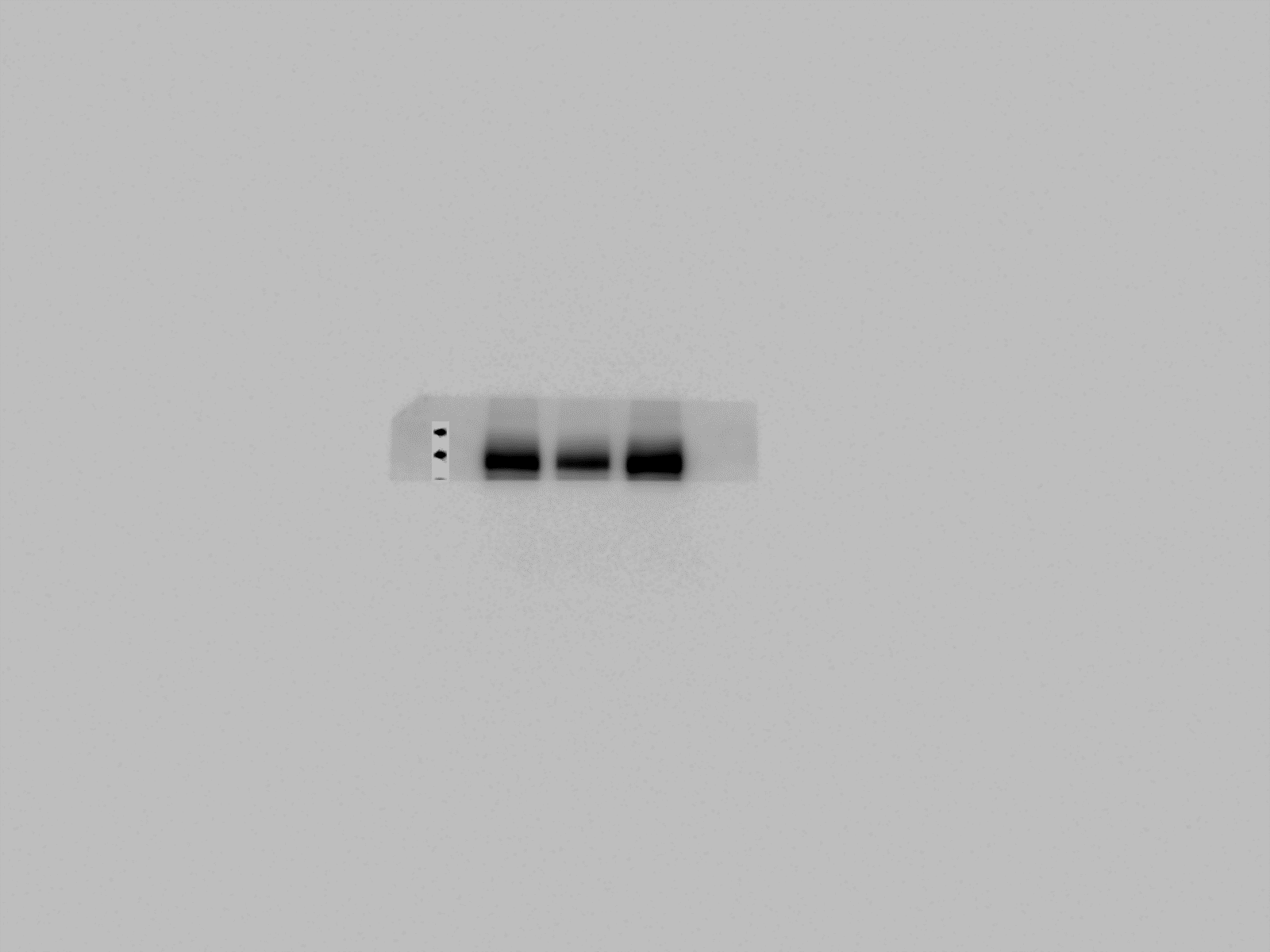


β-catenin


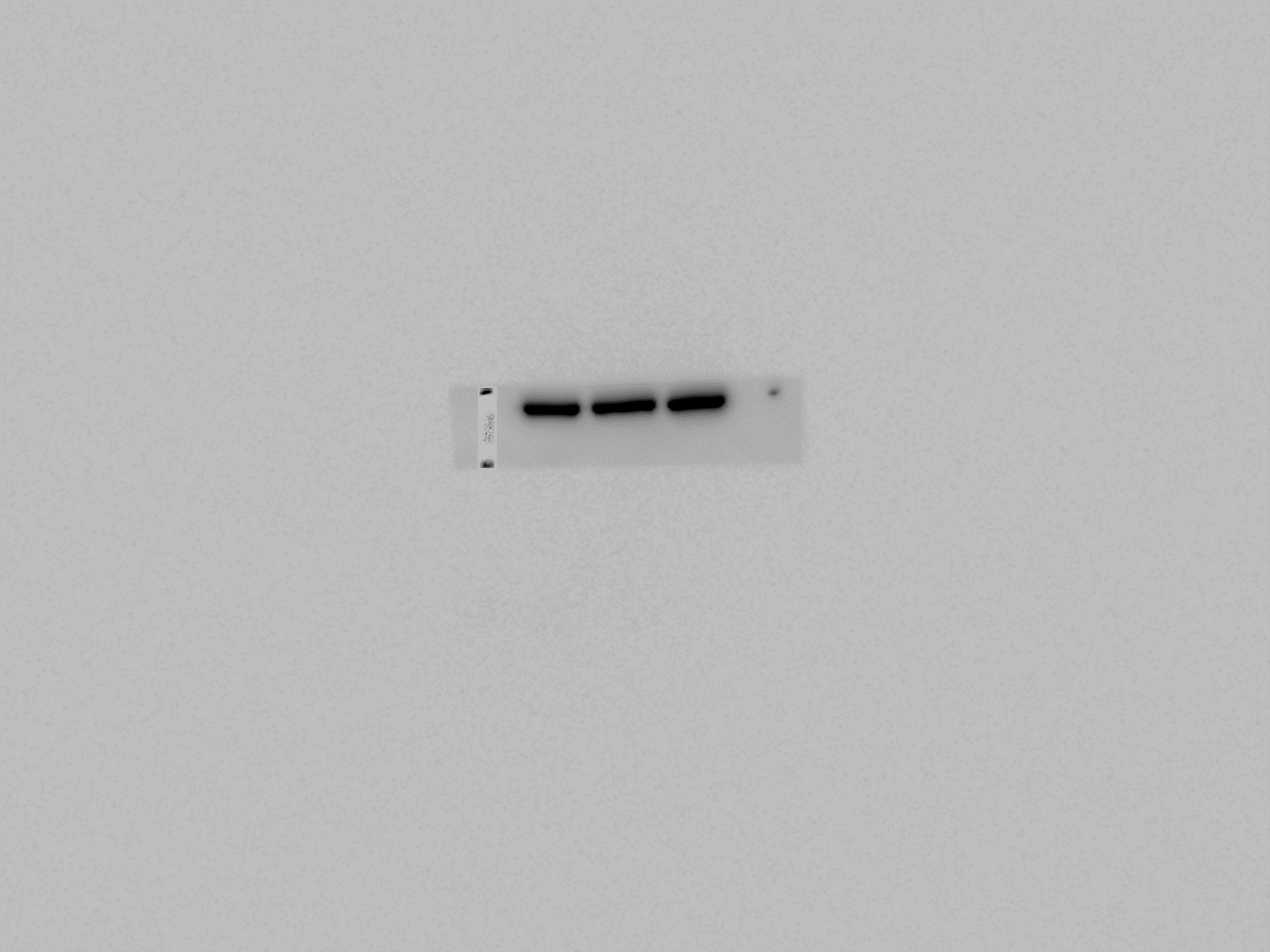


α-tubulin
