# Supplementary material for: MicroRNA-200c inhibits epithelial-mesenchymal transition, invasion, and migration of lung cancer by targeting HMGB1
Source: PLoS One. 2017 Jul 20;12(7):e0180844. doi: 10.1371/journal.pone.0180844 (PMC5519074; doi:10.1371/journal.pone.0180844)
Supplement: S5 Fig — S5 is Fig 9C and 9D raw data. (DOCX) [file pone.0180844.s005.docx]

**S5 Fig. Overexpression of HMGB1 increases the EMT of NSCLC xenografts in vivo.**

**Figure-9C**


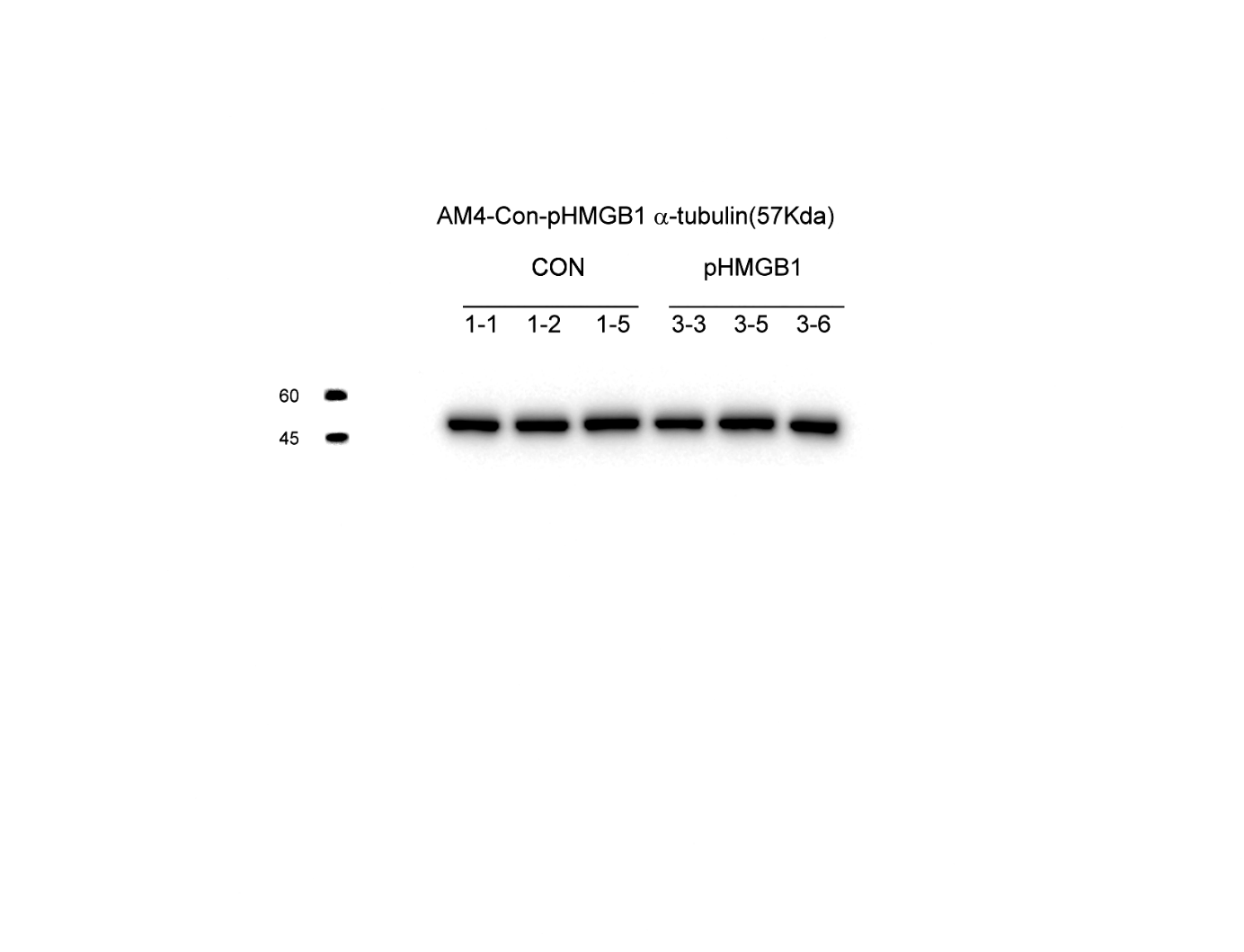


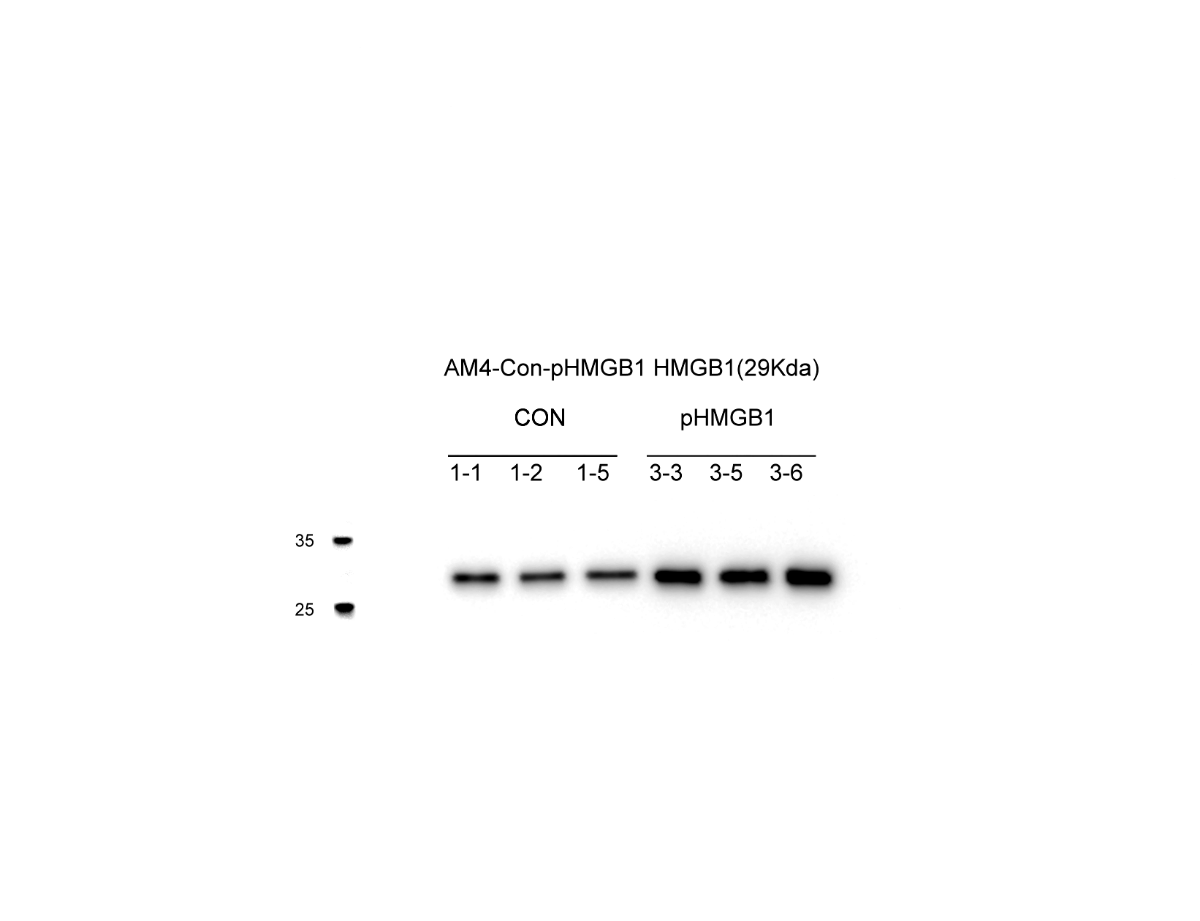


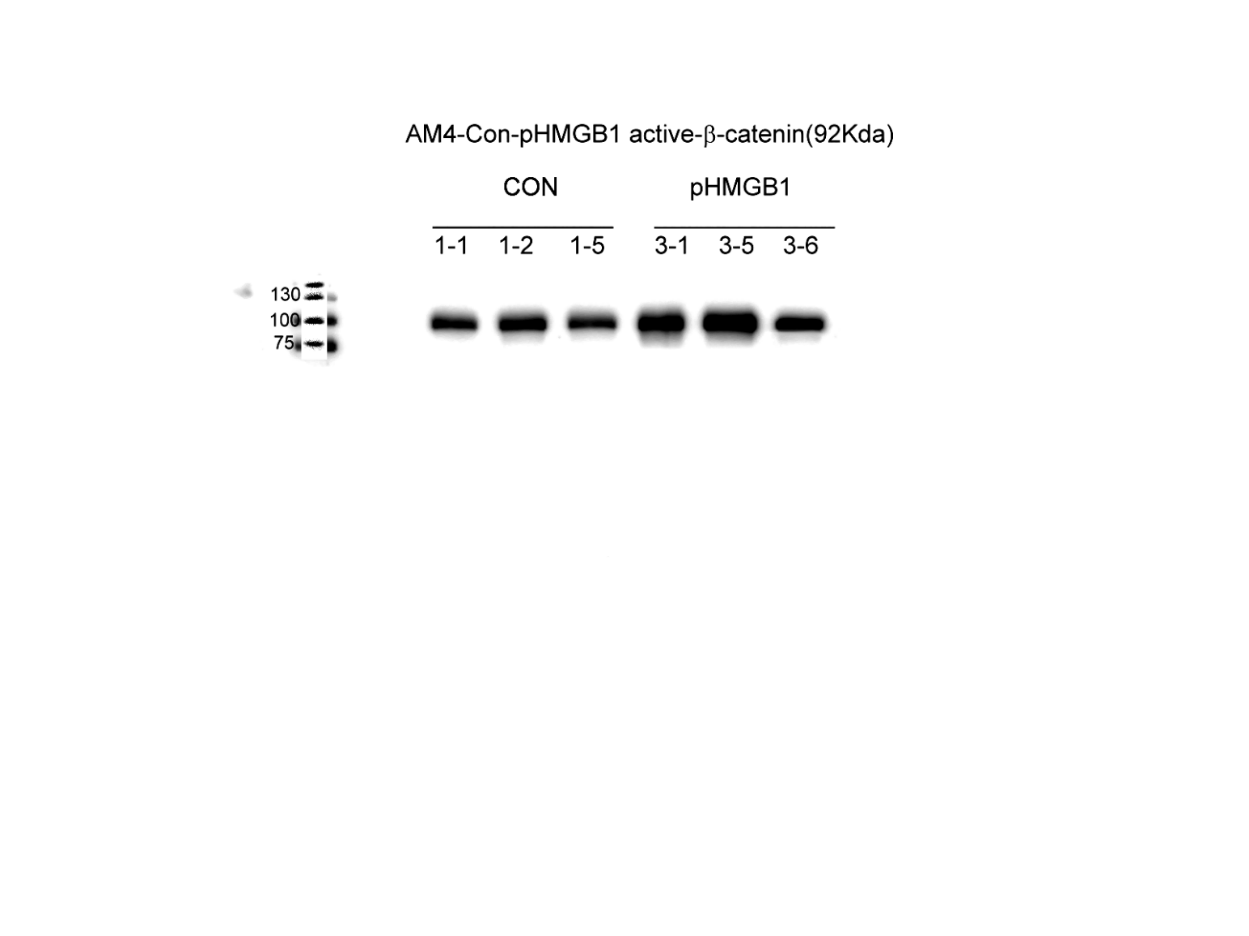


**Figure-9D**


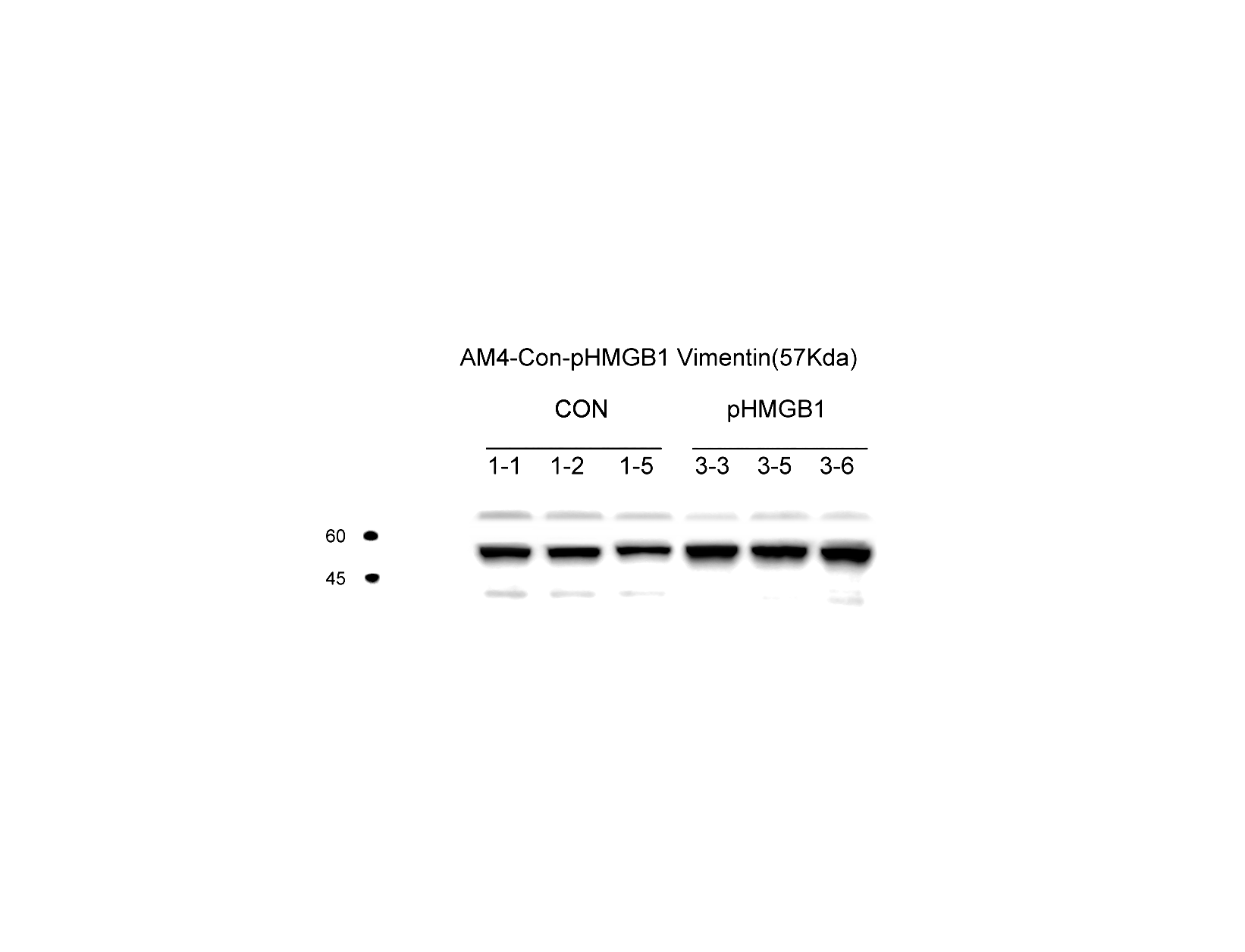


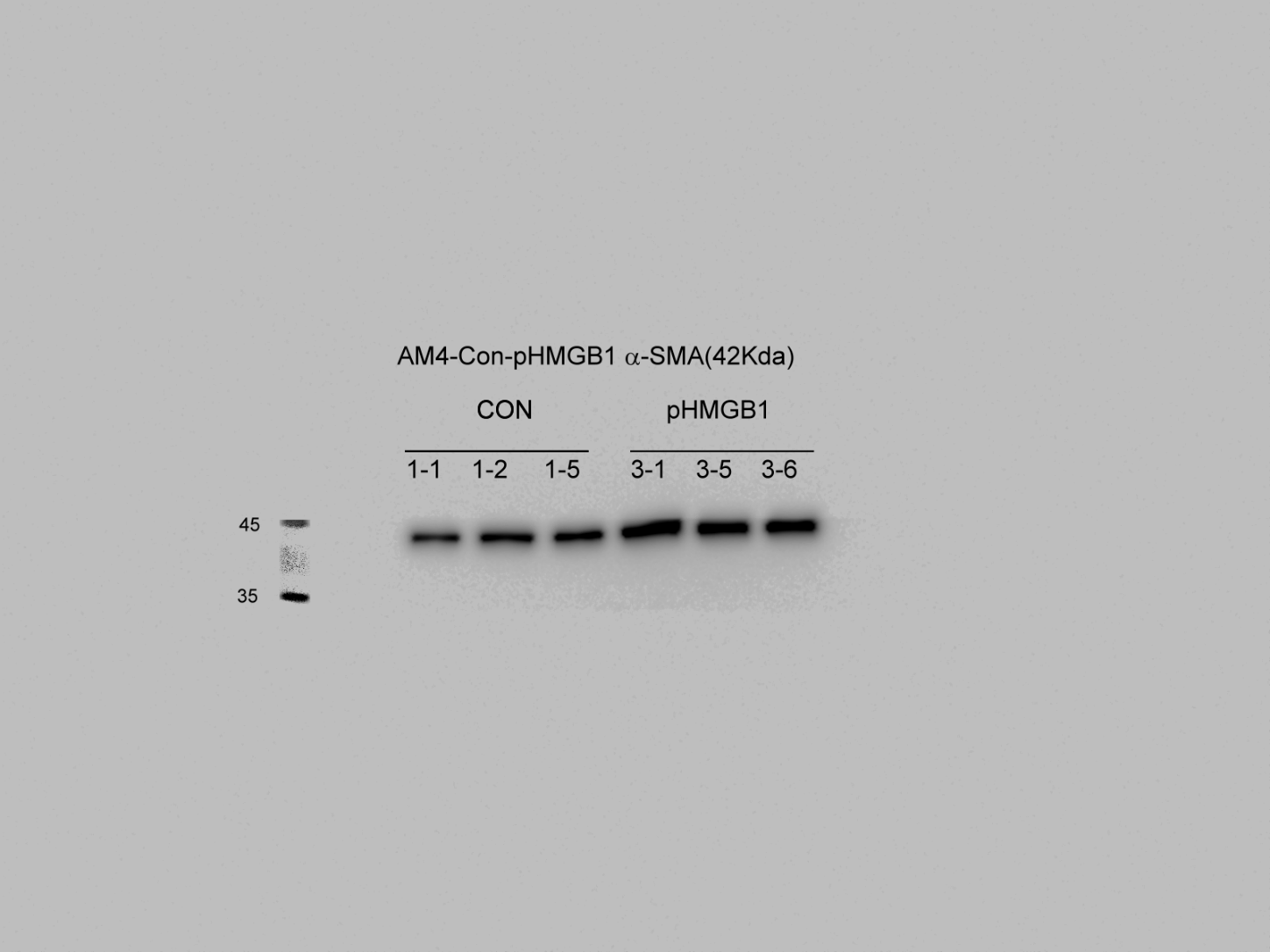


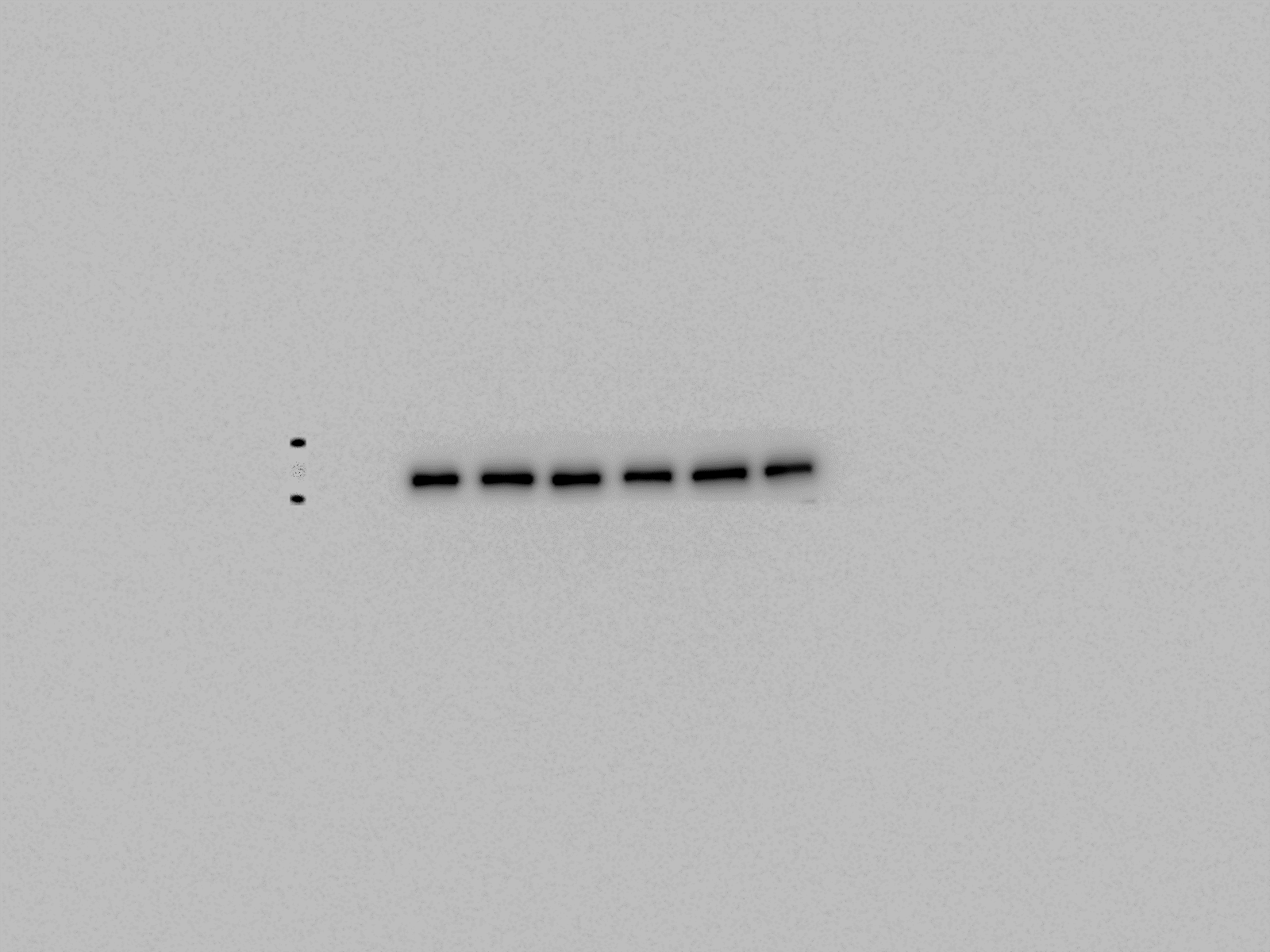


α-tubulin
